# New imidazolidindionedioximes and their Pt(II) complexes: synthesis and investigation of their antitumoral activities on breast cancer cells

**DOI:** 10.55730/1300-0527.3681

**Published:** 2024-01-22

**Authors:** Emrah KARAHAN, Tuğba GENÇOĞLU KATMERLİKAYA, Emel ÖNAL, Aydan DAĞ, Ayşe Gül GÜREK, Vefa AHSEN

**Affiliations:** 1 Department of Chemistry, Gebze Technical University, Kocaeli, Turkiye; 2 Department of Biotechnology, Institute of Health Sciences, Bezmiâlem Vakıf University, İstanbul, Turkiye; 3 Faculty of Engineering, Doğuş University, İstanbul, Turkiye; 4 Department of Pharmaceutical Chemistry, Faculty of Pharmacy, Bezmiâlem Vakıf University, İstanbul, Turkiye; 5 Pharmaceutical Application and Research Center, Bezmiâlem Vakıf University, İstanbul, Turkiye

**Keywords:** Imidazolidine, *vic*-dioxime, platinum complexes, breast cancer, cisplatin

## Abstract

Breast cancer is one of the most common types of cancer worldwide and has the most lethality ratio for females among all cancers. Although current cancer therapeutics have made considerable advancements, there is still room for improvement in terms of efficacy. Many anticancer drugs have a risk of causing serious adverse effects due to their nonspecific cytotoxic effects on both tumor and healthy cells. New therapeutics might have a greater ability to kill cancer cells, reduce the volume of tumors, and improve overall therapy response rates. Herein, we report the efficient synthesis and characterization of three amphi *vic*-dioximes and their six novel mono-, which are extremely rare in platinum chemistry, and bisplatinum(II) complexes for breast cancer treatment. Antitumoral activities of Pt(II) complexes have been investigated on CCD-1079Sk healthy fibroblast cell line, MCF-7 and MDA-MB-231 human breast cancer cell lines. Cytotoxicity, cell cycle, and apoptotic assays were performed. All new Pt(II) complexes exhibited selective antiproliferative effects on breast cancer cells by showing less cytotoxicity to healthy cells than known anticancer drugs cisplatin and bicalutamide. In vitro studies show that these new Pt complexes have high anticancer and antiproliferative effects and may be new alternatives to existing anticancer drugs.

## 1. Introduction

Cancer is the second cause of death worldwide after cardiovascular diseases and is responsible for nearly one in six deaths. Among all cancer types, lung and breast cancer have the highest mortality ratio globally for men and women, respectively [1].

At present, platinum-based anticancer drugs are the key treatment methods for neoplastic diseases. Cisplatin (cisplatinum or cis-diamminedichloroplatinum(II), cisPt) is a well-known metallodrug and widely used in the treatment of lung, breast, brain, ovarian, head and neck, prostate, and refractory non-Hodgkin’s lymphomas [2–4]. However, cisPt is a nonspecific chemotherapeutic drug that binds both healthy and cancerous cells, resulting in severe damage to both normal and neoplastic tissues [5,6]. Moreover, acquired or intrinsic drug resistance of cisPt is the foremost clinical impediment and limits the use of cisplatin in cancer therapy [7,10]. Based on first generation platinum drugs, second generation platinum drugs, namely carboplatin and nedaplatin, which have lower toxicities and are equivalent to cisPt, have been considered for development [11]. These drugs can be combined with paclitaxel and other drugs to treat lung cancer [12].

Like cisPt, carboplatin kills cancer cells by causing damage to DNA, has a nonspecific cell cycle, develops drug resistantance, and is a hindrance to cancer treatment. Currently, carboplatin is usually used synergistically with other drugs to enhance its therapeutic effect and avoid its drawbacks. Oxaliplatin, a third generation platinum drug, was the first platinum drug to which tumor cells resisted, preventing repair proteins from binding to DNA by incorporating the hydrophobic ligand cyclohexane diamine [13]. Oxaliplatin is the first platinum-based drug with significant efficacy in colon cancer. It has no crossresistance with cisplatin and carboplatin and has excellent therapeutic effects, although its drug interactions are not yet known. Lobaplatin, a third generation platinum drug, stands out for its lower toxicity compared to oxaliplatin. It possesses the unique ability to readily emulsify with other medications, resulting in the formation of oil-in-drug particles that can be effectively targeted and deposited in the affected area. Moreover, lobaplatin finds frequent application in transcatheter arterial chemoembolization due to its pH closely resembling the normal physiological pH of the human body. This characteristic enhances its compatibility and effectiveness in this particular treatment approach [14–17].

The development of platinum drugs across three generations has demonstrated their extensive utilization as crucial medications in the treatment of prevalent malignancies. On the other hand, platinum anticancer drugs generally lead to systemic toxicity and caused some severe adverse effects including nephrotoxicity, neurotoxicity and ototoxicity (irreversible hearing loss) [2,18–20]. These drawbacks encourage the scientists to reveal new platinum complexes which have better therapeutic efficacy and limited side effects on healthy cells.

In organic synthesis, oximes and their derivatives are commonly used as key intermediates for the preparation of a variety of heterocycles [21]. The oxime group acts as a ligand and forms complexes through the nitrogen and/or oxygen atom(s) in its structure. In the majority of complexes, the coordination bond is formed with the nitrogen atom [22]. Oxime compounds have been investigated for decades because of their significant roles as acetylcholinesterase reactivators and their use as therapeutics for a number of diseases [23–25].

Oximes have been reported to demonstrate diverse biological effects, encompassing antiinflammatory properties [26–28] as well as the ability to act as antihuman immunodeficiency (HIV) agents by inhibiting HIV protease [29,30]. On the other hand, incorporating an oxime group into a suitable chemical backbone is a viable strategy for developing cytotoxic agents, and numerous derivatives of oximes demonstrate therapeutic potential in cancer treatment [31–35] and the management of neurodegenerative disorders [36,37].

Oximes and their metal complexes are currently a topic of interest due to their diverse physical and chemical properties, reactivity, and potential applications in various chemical processes such as medicine, bioorganic systems, catalysis, electrochemical and electrooptical sensors [38–43]. *Vic*-Dioximes are another important ligand for metal complexes due to their bidentate coordination property. Due to the exceptional stability of vicinal dioxime, complexes derived from it have found wide-ranging applications, serving as effective tools in various fields. These applications include acting as a nonbiological substitute for coenzyme B12, facilitating metal analysis, enabling hydrogen production, etc. [44–49].

On the other hand, *vic*-dioximes are important complexing ligands that have received considerable attention in biology and chemistry. The ability of oxime ligands to stabilize particular metal ion redox states is important in bioinorganic systems. Babahan et al. reported that *vic*-dioxime complexes have an anticancer effect. They synthesized some dioxime derivatives containing thiosemicarbazone units and tested their complexes in vitro with Co(II) and Ni(II), on leukemia (HL-60 promyelocytic leukaemia) and colon cancer (HT-29 human colorectal adenocarcinoma) cell line. Both complexes showed apoptosis and necrosis in HT-29, with greater than 60% and 65% apoptosis in the HL-60 cell line, respectively [50]. Another study showed that the synthesis of a series of anthracene-9,10-dionedioxime compounds as biologically active prodrugs and used to treat cancer and other diseases [51]. For example (2,7-bis(((3R,5S)-3,5-dimethylpiperidin-1-yl) sulfonyl) anthracene-9,10-dionedioxime) showed a potent inhibition of β-catenin pathway [52,53]. The in vitro and in vivo studies showed inhibition of the growth of several cancer cell lines; therefore, it may be used as a therapeutic approach for cancer.

While *vic*-dioximes share a molecular structure similarity with oximes, there is a lack of biological studies concerning *vic*-dioximes in the existing literature. However, the available literature provides substantial information about oximes, including their effectiveness against different cancer cells and the impact of cis-platinum drugs on cancer treatment. As a result, novel Pt(II) (*vic*-dioxime) complexes have been synthesized to explore their biological properties and address the current gap in the literature [54–57].

Imidazolidine derivatives exhibit a wide range of structural possibilities, making them versatile molecules. These compounds play a crucial role in maintaining DNA stability and regulating the progression of the cell cycle. The heterocyclic core of these derivatives facilitates direct interaction with DNA, effectively controlling the process of DNA replication. Consequently, researchers have developed various derivatives with diverse biological properties, including anticancer, antibacterial, antifungal, antiviral, and antiinflammatory activities [58]. The imidazolidine ring has gained significant attention as a potential chemotherapeutic agent and has been extensively studied [59–61]. Its derivatives, including nilutamide, have been successfully commercialized and utilized as an effective treatment for numerous prostate cancer patients [62].

Superior biological activities have been observed in dioxime derivatives of imidazolidine [63,64]. In 1985, the first synthesis of the dioxime derivative of the imidazolidine ligand was accomplished [45] and no further synthesis of new imidazolidine dioxime derivatives has been reported since then. Recently, Eroğlu Gülümsek et al. successfully synthesized a Pt(II) complex containing aromatic amine groups from an N,N’-bis(aniline)glyoxime derivative [65]. This novel platinum complex exhibited promising anticancer activity in HCC cells. As known, cisPt and other platinum drugs exert their cytotoxicity by reacting with purine residues in DNA strand [66]. The mode of action starts with the cellular uptake of platinum complex and is followed by the dissociation of Cl^−^ ions which determine the rate of hydration to form a reactive platinum complex [67].

**Figure 1:**
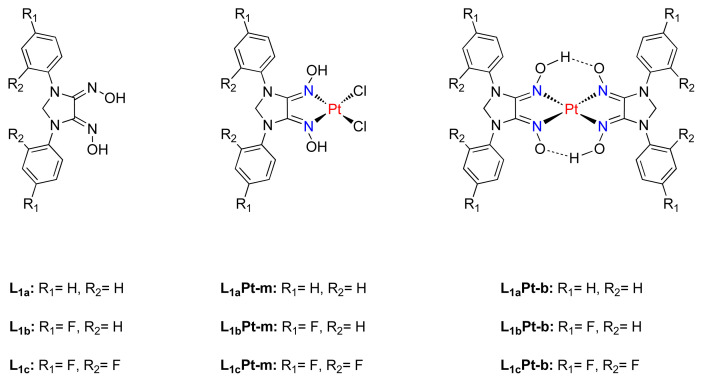
The structure of a series of imidazolidindionedioximes and their mono- and bis-Pt(II) complexes.

Taking into account these result and the limited number of research conducted on the utilization of highly potent imidazolidine dioxime ligands, we firstly designed a new series of imidazolidindionedioximes (L_1a_, L_1b_, L_1c_) (Figure 1). Considering well-defined action mechanism of platinum drugs, their mono-(L_1a_Pt-m, L_1b_Pt-m, L_1c_Pt-m) and bis-(L_1a_Pt-b, L_1b_Pt-b, L_1c_Pt-b) platinum complexes were synthesized. After that, in vitro cytotoxic effects of six new Pt(II) complexes were investigated on CCD-1079Sk healthy fibroblast cell line, as well as the MCF-7 and MDA-MB-231 human breast cancer cell lines.

## 2. Experimental

### 2.1. Materials and methods

Infrared spectra were recorded using a Perkin Elmer FT-IR System Spectrum BX spectrometer with an attenuated total reflection (ATR) accessory featuring a zinc selenide (ZnSe) crystal. MALDI-TOF MS mass spectrometry analyses were carried out on a Bruker Microflex LT MALDI-TOF MS spectrometer using 2,5-dihydroxybenzoic acid (DBH) or dithranol (DIT) as a matrix. NMR general characterization was conducted using a Bruker BioSpin AG Avance 500 mHz spectrometer (^1^H (500 MHz), ^13^C (125 MHz)). Samples were analyzed in the solvents of CDCl_3_ and DMSO-*d_6_*. All chemical shifts are stated in ppm (δ) relative to Si(CH_3_)_4_ as internal standard (δ = 0 ppm), referenced to the chemical shifts of residual solvent resonances (^1^H and ^13^C). Elemental analysis was carried out using a Thermo Scientific FLASH 2000 CHNS/O Analyzers instrument. All reagents and solvents were reagent-grade quality, were obtained from commercial suppliers. (*E,E*)-Dichloroglyoxime (DCGO) was prepared according to a described procedure [68].

^1^H and ^13^C NMR, FT-IR, MALDI-TOF MS and X-Ray diffractometer techniques were used to verify the proposed structures (see supplementary section, Figures S1–S41).

### 2.2. Synthesis and characterization

#### 2.2.1. Synthesis of diphenylmethanediamines (DMD)

Diphenylmethanediamines were synthesized according to literature [69]. Aniline (1a, 1b, 1c) (75 mmol) was dissolved in ethanol (15 mL), subsequently KOH (90 mmol) was added and heated to 80 °C. An aqueous solution of formaldehyde (34.5 wt.%, 3.0 mL, 37.5 mmol) was added over 5 min to a stirred solution of aniline in ethanol using a dropping funnel, and the mixture was stirred 3 h with a condenser. After completion of the reaction, two phases were separated with a separatory funnel and filtered over filter paper to a wide mouthed beaker and allowed to crystallize for 7–10 days. Crystals were filtered, washed with cold ethanol and dried under vacuum to yield diphenylmethanediamines (DMD) (2a, 2b, 2c).

2a: MP: 65–67 °C. Yield: 68% (5.06 g). Anal. calc for C_13_H_14_N_2_: C, 78.75; H, 7.12; N, 14.13%, found: C, 78.62; H, 7.15; N, 14.01. FT-IR (ATR): *ν*_max_, cm^−1^ = 3414, 3371, 3051, 2883, 1596, 1514, 1500, 1470, 1419, 1330, 1305, 1270, 1255, 1242, 1182, 1094, 1060, 880, 872, 748, 692. ^1^H NMR (500 MHz, DMSO-*d_6_*) δ_H_, ppm 7.06 (t, 4H, *J* = 7.9 Hz, CH), 6.67 (d, 4H, *J* = 7.9 Hz, CH), 6.54 (t, 2H, *J* = 7.2 Hz, CH), 6.24 (t, 2H, *J* = 5.6 Hz, −NH), 4.45 (t, 2H, *J* = 5.7 Hz, CH_2_). ^13^C NMR (125 MHz, DMSO-*d_6_*) δ_C_, ppm 147.7, 128.8, 116.0, 112.4, 52.5. MALDI-MS (m/z) calc 198.1 for C_13_H_14_N_2_; found: 198.5 [M]^+^, 221.5 [M+Na]^+^, 312.6 [cyclic 2a-3H] ^+^, 410.7 [M+Na+CHCA]^+^.

2b: MP: 74–76 °C. Yield: 66% (5.80 g). Anal. calc for C_13_H_12_F_2_N_2_: C, 66.66; H, 5.16; N, 11.96%, found: C, 66.77; H, 5.13; N, 12.03. FT-IR (ATR): *ν*_max_, cm^−1^ = 3406, 3352, 3048, 2919, 2874, 1861, 1610, 1504, 1455, 1420, 1378, 1298, 1249, 1202, 1127, 1093, 1048, 819, 738. ^1^H NMR (500 MHz, DMSO-*d_6_*) δ_H_, ppm 6.91 (t, 4H, *J* = 8.9 Hz, CH), 6.65 (dd, 4H, ^3^*J*_H-H_ = 9.0 Hz, ^4^*J*_H-F_ = 4.6 Hz, CH), 6.20 (t, 2H, *J* = 5.7 Hz, −NH), 4.40 (t, 2H, *J* = 5.7 Hz, CH_2_). ^13^C NMR (125 MHz, DMSO-*d_6_*) δ_C_, ppm 154.5 (d, ^1^*J_C-F_* = 231 Hz), 145.1 (d, ^4^*J_C-F_* = 2.2 Hz), 115.2 (d, ^2^*J_C-F_* = 21.9 Hz), 113.2 (d, ^3^*J_C-F_* = 7.2 Hz), 53.4. MALDI-MS (m/z) calc 369.1 for C_21_H_18_F_3_N_3_, cyclic form; found: 369.0 [M]^+^.

2c: MP: 80–82 °C. Yield: 41% (4.15 g). Anal. calc for C_13_H_10_F_4_N_2_: C, 57.78; H, 3.73; N, 10.37%, found: C, 57.83; H, 3.77; N, 10.29. FT-IR (ATR): *ν*_max_, cm^−1^ = 3449, 3085, 2940, 1827, 1602, 1514, 1456 1432, 1396, 1321, 1262, 1198, 1143, 1117, 1080, 1044, 953, 847, 791, 716. ^1^H NMR (500 MHz, DMSO-*d_6_*) δ_H_, ppm 7.10 (td, 2H, ^3^*J_H-H_* = 9.5 Hz, ^4^*J_H-F_* = 5.8 Hz, CH), 7.04 (ddd, 2H, ^3^*J_H-H_* = 11.7 Hz, ^3^*J_H-F_* = 9.0 Hz, ^4^*J_H-H_* = 2.6 Hz, CH), 6.86 (t, 2H, ^3^*J_H-F_* = 8.3 Hz, CH), 6.14 (bt, 2H, NH), 4.55 (t, 2H, *J* = 5.63 Hz, CH_2_). ^13^C NMR (125 MHz, DMSO-*d_6_*) δ_C_, ppm 153.1 (dd, ^1^*J_C-F_* = 234 Hz, ^3^*J_C-F_* = 11.1 Hz), 149.9 (dd, ^1^*J_C-F_* = 241 Hz, ^3^*J_C-F_* = 11.9 Hz), 132.2 (dd, ^2^*J_C-F_* = 11.6 Hz, ^4^*J_C-F_* = 2.4 Hz), 112.8 (dd, ^3^*J_C-F_* = 5.1, ^3^*J_C-F_*=8.5 Hz), 110.6 (dd, ^2^*J_C-F_* = 21.1, ^4^*J_C-F_*=3.4 Hz), 103.3 (dd, ^2^*J_C-F_* = 23.0, ^2^*J_C-F_* = 26.6 Hz), 52.3. MALDI-MS (m/z) calc 423.1 for C_21_H_15_F_6_N_3_, cyclic form; found: 420.2 [M-3H]^+^.

#### 2.2.2. Synthesis of *vic*-dioximes

Diphenylmethanediamines (DMD) (20 mmol) were dissolved in ethanol (100 mL), subsequently NaHCO_3_ was added and mixed for 5 min. A solution of dichloroglyoxime (DCGO) in ethanol (35 mL) was added over 30 min to a stirred suspension of DMD in ethanol using a dropping funnel, and the mixture was stirred for 3 h. The progress of the reaction was monitored by TLC. After completion of the reaction, precipitated particles were filtered and washed with water (100 mL) three times in a beaker to remove the remaining sodium bicarbonate. The crude product was washed with cold ethanol and diethyl ether, respectively, and dried under vacuum to yield *vic*-dioximes.

L_1a_: MP: 169–171 °C (dec.). Yield: 53% (2.99 g). Anal. calc for C_15_H_14_N_4_O_2_: C, 63.82; H, 5.00; N, 19.85%, found: C, 63.68; H, 4.95; N, 19.96. FT-IR (ATR): *ν*_max_, cm^−1^ = 3347, 3062, 2660, 1672, 1596, 1553, 1496, 1411, 1361, 1221, 956, 900, 804, 765, 730, 687. ^1^H NMR (500 MHz, DMSO-*d_6_*) δ_H_, ppm 10.70 (s, 1H, N-OH), 8.90 (bs, 1H, N-OH), 7.32 (t, 2H, *J* = 7.9 Hz), 7.22 (t, 2H, *J* = 7.9 Hz), 7.09 (t, 1H, *J* = 7.4 Hz), 7.03 (d, 2H, *J* = 7.9 Hz), 7.01 (d, 2H, *J* = 7.9 Hz), 6.92 (t, 2H, *J* = 7.3 Hz), 5.32 (s, 2H, CH_2_). ^13^C NMR (125 MHz, DMSO-*d_6_*) δ_C_, ppm 141.3, 140.9, 139.0, 134.6, 128.0, 127.9, 123.6, 121.9, 121.5, 119.9, 75.9. MALDI-MS (m/z) calc 282.1 for C_15_H_14_N_4_O_2_; found: 282.1 [M]^+^.

L_1b_: MP: 213–215 °C (dec.). Yield: 64% (4.07 g). Anal. calc for C_15_H_12_F_2_N_4_O_2_: C, 56.60; H, 3.80; N, 17.60%, found: C, 56.71; H, 3.87; N, 17.47. FT-IR (ATR): *ν*_max_, cm^−1^ = 3381, 3089, 2770, 1664, 1638, 1506, 1456, 1409, 1368, 1205, 968, 946, 826, 816, 794, 668. ^1^H NMR (500 MHz, DMSO-*d_6_*) δ_H_, ppm 10.60 (s, 1H, N-OH), 8.93 (bs, 1H, N-OH), 7.16–7.08 (m, 4H, CH), 7.05–7.03 (m, 4H, CH), 5.27 (s, 2H, CH_2_). ^13^C NMR (125 MHz, DMSO-*d_6_*) δ_C_, ppm 158.9 (d, ^1^*J_C-F_* = 241 Hz), 157.8 (d, ^1^*J_C-F_* = 238 Hz), 141.1 (s, C-F_4_), 137.7 (d, ^4^*J_C-F_* = 2.5 Hz), 135.2, 134.7, 124.3 (d, ^3^*J_C-F_* = 8.3 Hz), 122.0 (d, ^3^*J_C-F_* = 7.8 Hz), 114.6 (d, ^2^*J_C-F_* = 22.6 Hz), 114.4 (d, ^2^*J_C-F_* = 22.4 Hz), 75.9. MALDI-MS (m/z) calc 318.1 for C_15_H_12_F_2_N_4_O_2_; found: 318.1 [M]^+^.

L_1c_: MP: 246–248 °C (dec.). Yield: 31% (2.20 g). Anal. calc for C_15_H_10_F_4_N_4_O_2_: C, 50.86; H, 2.85; N, 15.82%, found: C, 50.95; H, 2.81; N, 15.88. FT-IR (ATR): *ν*_max_, cm^−1^ = 3360, 3094, 2689, 1679, 1609, 1554, 1509, 1406, 1362, 1270, 1245, 1220, 1210, 1181, 1145, 1090, 1068, 972, 965, 956, 889, 847, 722. ^1^H NMR (500 MHz, DMSO-*d_6_*) δ_H_, ppm 10.34 (s, 1H, N-OH), 8.88 (s, 1H, N-OH), 7.32–7.26 (m, 2H, CH), 7.23–7.17 (m, 2H, CH), 7.05 (td, 1H, *J* = 8.5 Hz, *J* = 1.9 Hz, CH), 6.98 (td, 1H, *J* = 8.5 Hz, *J* = 1.9 Hz, CH), 5.16 (s, 2H, CH_2_). ^13^C NMR (125 MHz, DMSO-*d_6_*) δ_C_, ppm 159.8 (dd, ^1^*J_C-F_* = 245 Hz, ^3^*J_C-F_* = 11.5 Hz), 158.8 (dd, ^1^*J_C-F_* = 242 Hz, ^3^*J_C-F_* = 11.0 Hz), 156.5 (dd, ^1^*J_C-F_* = 249 Hz, ^3^*J_C-F_* = 12.5 Hz), 155.5 (dd, ^1^*J_C-F_* = 247 Hz, ^3^*J_C-F_* = 13.0 Hz), 141.0, 136.2, 127.9 (dd, ^2^*J_C-F_* = 10.0 Hz, ^4^*J_C-F_* = 1.5 Hz), 126.6 (d, ^3^*J_C-F_* = 8.6 Hz), 126.1 (dd, ^2^*J_C-F_* = 12.2 Hz, ^4^*J_C-F_* = 3.7 Hz), 124.1 (d, ^3^*J_C-F_* = 7.5), 110.7 (dd, ^2^*J_C-F_* = 18.8, ^4^*J_C-F_* = 3.1 Hz), 110.6 (dd, ^2^*J_C-F_* = 17.8, ^4^*J_C-F_* = 2.7 Hz), 104.0 (dd, ^2^*J_C-F_* = 26.7, ^2^*J_C-F_* = 23.9 Hz), 103.9 (dd, ^2^*J_C-F_* = 26.6, ^2^*J_C-F_* = 22.7 Hz), 74.7. MALDI-MS (m/z) calc 354.1 for C_15_H_10_F_4_N_4_O_2_; found: 354.9 [M]^+^.

#### 2.2.3. Synthesis of monoplatinum complexes

To a sonicated and filtered solution of PtCl_2_ (266.0 mg, 1 mmol) in DMSO (20 mL), a solution of the dioxime (L_1a_, L_1b_, L_1c_) (1 mmol) in DMSO (10 mL) was added dropwise within 5 min. The mixture was then stirred at 40 °C for 2 h. After the completion of reaction, the mixture was cooled to room temperature. Then, water (20 mL) and brine (40 mL) were added to precipitate formed complexes. The resulting suspension was centrifuged, and solid particles were subsequently washed with water, ethanol, and diethyl ether, respectively, and dried in vacuo to yield greenish-brownish monoplatinum complexes.

L_1a_Pt-m: MP: 185–187 °C (dec.). Yield**:** 28% (166 mg). Anal. calc for C_15_H_14_Cl_2_N_4_O_2_Pt: C, 32.86; H, 2.57; N, 10.22%, found: C, 32.72; H, 2.58; N, 10.37. FT-IR (ATR): *ν*_max_, cm^−1^ = 3072, 2914, 1674, 1592, 1492, 1445, 1209, 1163, 1072, 102, 1006, 921, 754, 737, 688. ESI-MS (m/z) calc 546.0 for C_15_H_14_Cl_2_N_4_O_2_Pt; found: 589.1 [M-Cl+DMSO]^+^.

L_1b_Pt-m: MP: 160–162 °C (dec.). Yield**:** 31% (193 mg). Anal. calc for C_15_H_12_Cl_2_F_2_N_4_O_2_Pt: C, 30.84; H, 2.07; N, 9.59%, found: C, 30.93; H, 2.08; N, 9.69. FT-IR (ATR): *ν*_max_, cm^−1^ = 3007, 1631, 1502, 1411, 1295, 1213, 1154, 1092, 1013, 923, 829, 759, 693. ESI-MS (m/z) calc 582.0 for C_15_H_12_Cl_2_F_2_N_4_O_2_Pt; found: 625.1 [M-Cl+DMSO]^+^.

L_1c_Pt-m: MP: 140–142 °C (dec.). Yield**:** 53% (349 mg). Anal. calc for C_15_H_10_Cl_2_F_4_N_4_O_2_Pt: C, 29,05; H, 1.63; N, 9.03%, found: C, 28.97; H, 1.66; N, 9.15. FT-IR (ATR): *ν*_max_, cm^−1^ = 2900, 2166, 1685, 1608, 1506, 1433, 1269, 1246, 1142, 1097, 1020, 965, 849, 733, 695. ESI-MS (m/z) calc 618.0 for C_15_H_10_Cl_2_F_4_N_4_O_2_Pt; found: 661.1 [M-Cl+DMSO]^+^.

#### 2.2.4. Synthesis of bisplatinum complexes

PtCl_2_ (199.50 mg, 0.75 mmol) was dissolved in DMSO (10 mL) and sonicated for 5 min. The resulting solution was filtered to remove insoluble particles and heated to 75 °C. To a stirred solution of oxime ligand (L_1a_, L_1b_, L_1c_) (1.5 mmol) in DMSO (20 mL), a preheated solution of PtCl_2_ in DMSO and an aqueous KOH solution (15 mL, 0.1 N) were added drop by drop at 75–80 °C, respectively. The reaction mixture was stirred for 30 min at this temperature and then allowed to warm to room temperature.

L_1a_Pt-b: During the cooling of the solution, precipitates were formed. The resulting suspension was centrifuged and washed 3 times with water. Crude platinum complexes were dissolved in dichloromethane and precipitated with hexane to yield the corresponding platinum complex. MP: >250 °C. Yield**:** 29% (162 mg). Anal. calc for C_30_H_26_N_8_O_4_Pt: C, 47.56; H, 3.46; N, 14.79%, found: C, 47.36; H, 3.28; N, 14.62. FT-IR (ATR): *ν*_max_, cm^−1^ = 3053, 1778, 1690, 1590, 1494, 1448, 1215, 1072, 1026, 832, 749, 691. MALDI-MS (m/z) calc 756.1 for C_30_H_26_N_8_O_4_Pt; found: 757.1 [M+H]^+^.

L_1b_Pt-b: During the cooling of the solution, precipitates were forming slowly. The resulting suspension was left at room temperature for 2 h, and subsequently was centrifuged and washed 3 times with water and dichloromethane to yield a corresponding platinum complex. MP: > 250 °C. Yield**:** 24% (149 mg). Anal. calc for C_30_H_22_F_4_N_8_O_4_Pt: C, 43.43; H, 2.67; N, 13.51%, found: C, 43.64; H, 2.77; N, 13.88. FT-IR (ATR): *ν*_max_, cm^−1^ = 3007, 1782, 1656, 1601, 1504, 1396, 1276, 1261, 1223, 1154, 885, 831, 765, 754. MALDI-MS (m/z) calc 828.1 for C_30_H_22_F_4_N_8_O_4_Pt; found: 829.6 [M+H]^+^.

L_1c_Pt-b: During the cooling of the solution, precipitates were forming very slowly. The resulting suspension was left at room temperature for two weeks, and subsequently was centrifuged and washed 3 times with water and dichloromethane and dried under vacuum to yield the corresponding platinum complex. MP: >250 °C. Yield**:** 18% (123 mg). Anal. calc for C_30_H_18_F_8_N_8_O_4_Pt: C, 39.97; H, 2.01; N, 12.43%, found: C, 39.80; H, 2.01; N, 12.70. FT-IR (ATR): *ν*_max_, cm^−1^ = 3113, 1600, 1503, 1432, 1267, 1140, 1096, 1019, 965, 846, 732. MALDI-MS (m/z) calc 900.1 for C_30_H_18_F_8_N_8_O_4_Pt; found: 901.1 [M+H]^+^.

### 2.3. In vitro studies

#### 2.3.1. Cell culture

CCD-1079Sk healthy fibroblast cell line, MCF-7, and MDA-MB-231 breast cancer cell lines were used in this study. The cells were grown in DMEM/F12 medium containing 10% fetal bovine serum (FBS) and 100 U/mL penicillin/streptomycin at 37 °C in a humidified incubator with 5% CO_2_. After bringing 80% confluency, the cells were detached using a trypsin solution. For further experiments, cells were resuspended in the growth medium after collection and centrifugation.

#### 2.3.2. MTT assay

To determine cytotoxicity, the MTT assay was performed using L_1a_Pt-m, L_1b_Pt-m, L_1c_Pt-m, L_1a_Pt-b, L_1b_Pt-b, L_1c_Pt-b, cisPt, and bicalutamide (bical) samples at 10 different concentrations ranging from 100 μM to 0.19 μM (100, 50, 25, 12.5, 6.25, 3.12, 1.56, 0.78, 0.39, and 0.19 μM). The samples were dissolved in DMSO and diluted with the medium. Initial DMSO concentration was kept below 1% to avoid toxicity from the solvent. Briefly, the cells were seeded to 96-well plates at 5 × 10^3^ cells/well in a volume of 200 μL, and cells were inoculated overnight. After that, old medium was removed, 100 μL of fresh medium and 100 μL of sample were added to the wells. The cells were incubated with the samples for 24 h. At the end of incubation with the samples, 30 μL of 3-(4,5-dimethylthiazol-2-yl)-2,5-diphenyltetrazolium bromide (MTT) solution was added. After 3 h the medium was removed and 100 μL of DMSO was added to dissolve the formazan crystals. Then the absorbance values were recorded at 540 nm using an ELISA microplate reader (Synergy H1, BioTek) [70]. All the experiments were carried out in triplicates, and the results were presented as a mean ± standard deviation. The IC_50_ values of samples were calculated using a percentage of relative cell viability via GraphPad Prism 5. The viability percentages of cells were calculated using the following formula:


%relative cell viability=(Abssample/Abscontrol)×100.

#### 2.3.3. Cell cycle assay

The cell cycle kit was used for the cell cycle experiment. Briefly, the cells were seeded into 12-well plates in a volume of 1 mL at 2 × 10^5^ cells/well, and cells were inoculated overnight. After that, 500 μL of fresh medium and 500 μL of the sample at the concentration of IC_50_ were added to the wells. The cells were incubated with the samples for 24 h, then detached using 0.5 mL of trypsin solution, cells were collected and centrifuged at 3000 g for 3 min. The collecting cells were washed with 1 mL of PBS and fixed with 1 mL of 70% cold ethanol at −20 °C for 24 h. After that, 200 μL of staining solution containing propidium iodide (PI) and RNAse reagents were added to the cells, and the cells were incubated for 30 min in the dark. Cell cycle profiles of synthesized compounds were analyzed using a flow cytometer (BD Accuri C6). The data were analyzed through BD software. Each assay was investigated in triplicate [71].

#### 2.3.4. Apoptosis assay

FITC-Annexin V/7-AAD kit was used for the apoptosis experiment. Briefly, the cells were seeded into 12-well plates at 2 × 10^5^ cells/well in 1 mL volume and cells were inoculated overnight. After that, 500 μL of fresh medium and 500 μL of sample at the concentration of IC_50_ were added to the wells. The cells were incubated with the samples for 24 h, then detached using 0.5 mL of trypsin solution, cells were collected and centrifuged at 3000 g for 3 min. The cells were washed with 200 μL of cell staining buffer, then dispersed in 100 μL of binding buffer. Following this, 5 μL of FITC-Annexin V and 10 μL of 7-AAD were added, and the mixture was incubated in the dark for 30 min. The samples were then analyzed by flow cytometry [71].

#### 2.3.5. Statistical analysis

The statistical analysis was performed using the IBM SPSS statistics version 22. One-way analysis of variance (ANOVA), followed by Tukey’s posthoc test for multiple comparisons and independent samples t-test for comparison between two groups, was performed with significance defined as p < 0.05.

## 3. Results and discussion

### 3.1. Synthesis and characterization

**Scheme 1:**
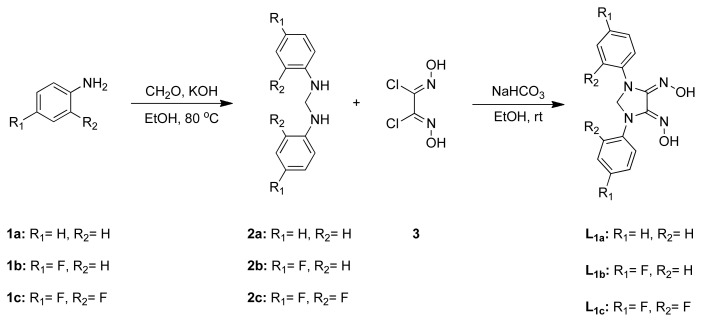
Synthesis of DMDs and *vic*-dioximes.

Diphenylmethanediamines (2a, 2b, 2c) (DMD) were obtained by the reaction of corresponding aniline with an aqueous solution of formaldehyde in ethanol under basic conditions at 80 °C (Scheme 1). It is known that the reaction between aniline and formaldehyde is highly pH-dependent [72–74]. Even when the desired products are isolated, they remain highly susceptible to cyclization or rearrangements based on the acidity or alkalinity of the medium. The purification of the generated DMDs (2a, 2b, 2c) was initially attempted using column chromatography. However, it was observed that even on TLC plates, all DMD derivatives underwent decomposition. As a result, 2a, 2b, and 2c were purified through crystallization by allowing the reaction mixture to stand at room temperature for an extended period.

**Figure 2:**
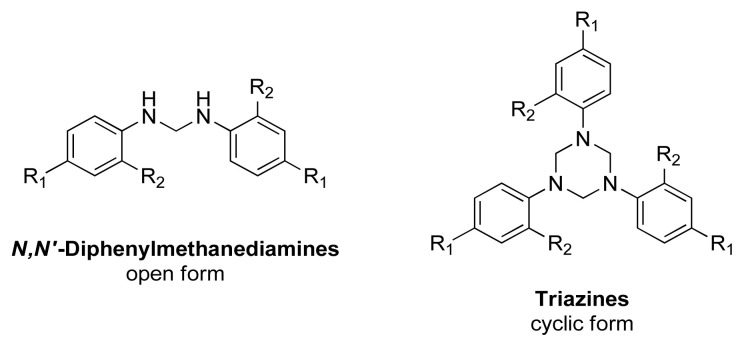
General structure of diphenymethanediamines and triazines.

DMDs have been characterized by NMR and CDCl_3_ is used as a common solvent. However, it is observed that all DMDs start to generate triazine, trimeric cyclic, structure in CDCl_3_ with a different rate. Especially, 2b is completely converted from methanediamine to triazine form in 30 min (Figure 2). Disappearance of 3400–3500 ν(N-H) stretching signal from IR spectra shows cyclization but NMR signals help us elucidate exact chemical structure easily via methylene bridge protons and loss of −NH signals (Figures S11–S13). NMR peak appears as singlet or triplet with respect to cyclic and open structure for −CH_2_ protons, respectively. Therefore, NMR analysis was performed in DMSO-*d_6_* and it is shown that diphenylmethanediamines (2a, 2b, 2c) are stable. Bridge protons resonate as a triplet due to adjacent −NH protons and this proves that DMDs (2a, 2b, 2c) have desired structures (Figures S3, S7, and S14). DMDs were also analyzed by MALDI-MS and all peaks were fully compatible with desired structures as described in Giumanini et al.’s study (Figures S6, S10, and S17) [72]. Diphenylmethanediamine (2a) was obtained as a colorless crystal, and it was also verified by X-ray analysis that DMD’s forms triclinic crystals (Figures S1 and S2; Table S).

The skeleton of *vic*-dioximes was created by the fusion of DMD derivatives with antidichloroglyoxime (DCGO) which is synthesized according to the literature (Scheme 1) [68]. It is expected to obtain anti*vic*-dioxime derivatives starting from anti-DCGO. However, the presence of one sharp singlet around 10.5 ppm and one broad singlet around 9.0 ppm, along with two distinct phenyl rings in the NMR spectrum, verifies that all oxime derivatives possess an amphi structure. The appearance of sharp IR peaks in the range of 1660–1680 and 950–970 also indicates the presence of ν(C=N) and ν(N-O) bonds for *vic*-dioximes, respectively. The mass spectrum of *vic*-dioximes shows molecular ion peaks [M]^+^ for all derivatives (L_1a_, L_1b_, L_1c_), confirming the formation of desired compounds (Figures S21, S25, and S29).

**Scheme 2:**
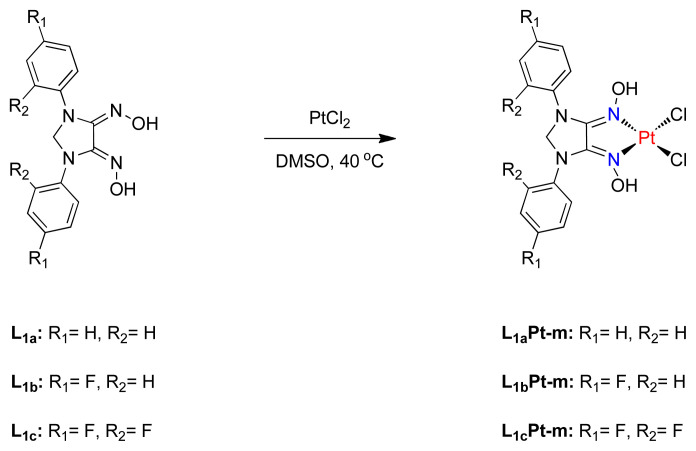
Synthesis of monoplatinum complexes.

Monoplatinum complexes (L_1a_Pt-m, L_1b_Pt-m, L_1c_Pt-m) were synthesized by mixing corresponding oxime with PtCl_2_ in 1:1 ratio in DMSO at 40 °C (Scheme 2). The reaction mixture was treated with brine to precipitate desired complexes, which will be characterized by mass spectroscopy. The loss of one chloride (Cl^−^) ion and addition of DMSO ligand ([M-Cl+DMSO]^+^) is the general feature of the prepared monoplatinum complexes, which are rarely found in platinum chemistry. The additional DMSO in the mass spectroscopy is the result of DMSO solvent used for the mass analysis.

**Scheme 3:**
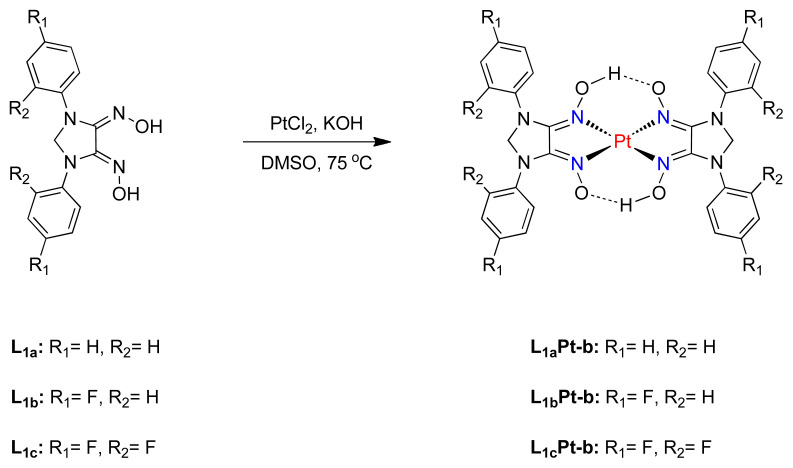
Synthesis of bisplatinum complexes.

Besides, bisplatinum complexes were created by changing metal-to-ligand ratio from 1:1 to 1:2 (Scheme 3). The reaction was performed with addition of ethanolic solution of KOH at 75–80 °C to favor biscomplex formation. It has been demonstrated that all bisplatinum complexes exhibit similar [M+H]^+^ ion peak in mass spectrometer analysis. All novel platinum(II) compounds were characterized by elemental analysis, IR and mass spectrometry and the results were fully compatible with mono- and bisplatinum complexes.

The disappearance of the broad ν(NO-H) peak around 2500–3000 and the sharp ν(N-O) peak around 950–970, as well as the shift of the ν(C=N) frequency, indicate the formation of the chelate ring through the nitrogen atom of the oxyimino groups of the ligand as expected, resulting in a square planar structure.

### 3.2. In vitro studies

**Figure 3:**
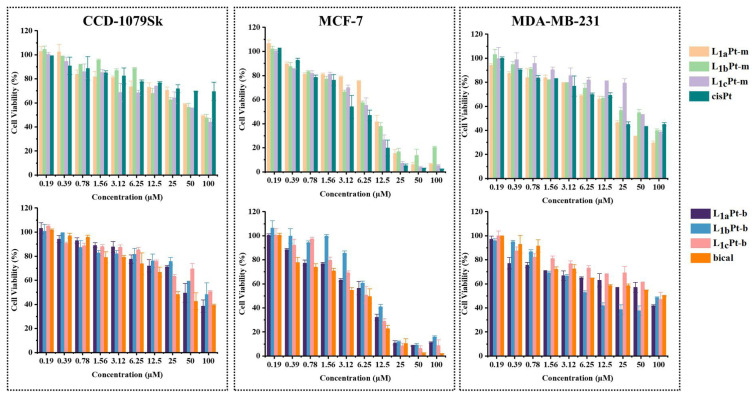
The cytotoxicity results of tested compounds, cisPt and bical.

In this study, in vitro cytotoxic effects of L_1a_Pt-m, L_1b_Pt-m, L_1c_Pt-m, L_1a_Pt-b, L_1b_Pt-b, L_1c_Pt-b were investigated on CCD-1079Sk healthy fibroblast cell line, MCF-7, and MDA-MB-231 human breast cancer cell lines at 10 different concentrations. The cytotoxicity results of compounds were compared with the cytotoxic effect of anticancer drug cisPt and bical on the same cell lines. The half inhibitory concentration (IC_50_) values were calculated based on these data. The cytotoxic effects of the compounds targeted against CCD-1079Sk, MCF-7, and MDA-MB-231 cell lines were expressed as relative cell viability (Figure 3), and IC_50_ values which are the compound concentrations (μM) that exhibited 50% cell viability (Table 1). These data show that compounds have higher lethality towards the MCF-7 and MDA-MB-231 cells compared to CCD-1079Sk cells. Statistically significant differences were observed in the cytotoxicity experiment of six compounds for CDD-1079Sk compared to MCF-7 and MDA-MB-231 breast cancer cells (p < 0.001).

The synthesized compounds show greater cytotoxicity to cancer cells than to healthy cells. These results show that the compounds have antiproliferative effects against breast cancer cell lines selectively. Therapeutic index (TI, TD_50_/ED_50_, the ratio of toxic dose–IC_50_ values of compounds on healthy cell line and the effective dose–IC_50_ values of compounds on cancer cell lines) is a measure of the selectivity of a compound. The TI of compounds is 2.74 and 1.64-fold or higher than TI of bical for MCF-7 and MDA-MB-231 cells, respectively. There are significant differences between the selectivity of tested compounds except L_1c_Pt-m and bical (p < 0.05).

**Figure 4:**
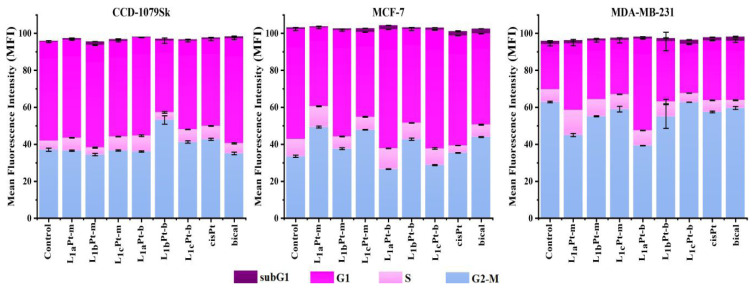
The cell cycle assay results of tested compounds, cisPt and bical for CCD-1079Sk, MCF-7, and MDA-MB-231 cell lines.

The compounds have a higher mortality rate in MCF-7 cells than in MDA-MB-231 cells. It is seen that cell viability is less than 20% at high doses (≥ 25 μM) used for MCF-7 cell lines. According to cytotoxicity results, compounds exhibit similar cytotoxicity to that of cisPt and bical used as a positive control group in both MCF-7 and MDA-MB-231 cells. In the literature, the IC_50_ values of cisPt and bical were found to be close to the values in our study [75–77]. The cytotoxicity of imidazolidine-derived compounds on the breast cancer cell line is consistent with the literature [58,59,61]. In the study of Bakalova et al., in which the imidazolidine derivative Pt complex was synthesized, the IC_50_ value of the complex in MDA-MB-231 cells was found to be 24.5 μM [78]. In another study, the IC_50_ value of biotin derived Pt complexes were found to be ≥ 32 μM for MCF-7 and ≥ 18 μM for MDA-MB-231 cells at 48 h [79]. In our study, the fact that the Pt-derived compounds with a minor IC_50_ value indicate that MDA-MB-231 cells were sensitive to the compounds. According to the literature, it is known that Pt complexes have an effect on gene expression by crosslinking with DNA and have anticancer activity, including breast cancer [80]. In the study of Cai et al., a new Pt complex was shown to have excellent anticancer activity against breast cancer cells, as well as to play a role in regulating the tumor microenvironment by inhibiting the JAK2/STAT3 pathway [81]. Bazsefidpar et al. reported that the Pt complexes they developed in their studies had a stronger antiproliferative and antineoplastic effect in vitro and in vivo compared to standard drugs (cisplatin, oxaliplatin, and carboplatin) [82]. Platinum complexes are important candidates for use in breast cancer treatment in preclinical and clinical studies due to their antiproliferative, antimigratory and prooxidative potential [83–87]. Uncontrolled proliferation due to the nonregulated cell cycle is the main cause of tumorigenesis. Investigation of the effect of compounds on the cell cycle attracts the attention of researchers in anticancer drug development. The cell cycle process could be affected by a disruption that prevents cancer cell proliferation. For this reason, the effects of compounds on cell cycle progression were investigated on CCD-1079Sk, MCF-7, and MDA-MB-231 cell lines through DNA flow cytometry (Figure S42). The results of the cell cycle assay demonstrated that treatment of the compounds to different cell lines led to cell arrest in various phases of the cell cycle. In the posttreatment, for compounds L_1a_Pt-m and L_1a_Pt-b, the MDA-MB-231 cell populations in the G1-phase increased to 36.1% and 49.6%, respectively, compared to 24.5% in control cells. Similarly, for compounds L_1a_Pt-b and L_1c_Pt-b, the MCF-7 cell populations in the G1-phase increased to 64.4% and 64.1%, respectively, compared to 59.5% in control cells (Figure 4).

**Figure 5:**
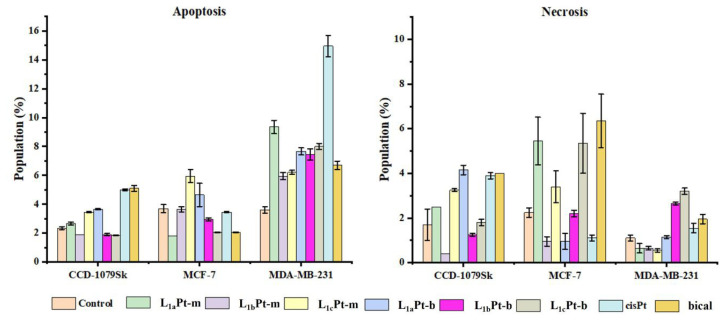
The apoptosis assay results of tested compounds, cisPt and bical for CCD-1079Sk, MCF-7, and MDA-MB-231 cell lines.

The Annexin V conjugated Alexa Fluor 488/7-AAD binding assay is based on the difference in fluorescence intensity between apoptotic and nonapoptotic cells stained with the fluorescent Annexin V/7-AAD, which is measured by flow cytometry. Therefore, an apoptosis assay was performed to determine the rate of live, apoptotic, and necrotic cells (Table 2, Figure S43). It is seen that compounds induce early and late apoptosis in MDA-MB-231 cells. Additionally, the treatment of compounds, especially L_1a_Pt-m, L_1c_Pt-m, and L_1c_Pt-b, induces more cancerous cells to undergo apoptosis, resulting in cell death for MCF-7 cells (Figure 5).

## 4. Conclusion

In summary, six novel platinum(II) complexes of imidazolidindionedioximes were created and assessed for their anticancer activity. The fact that the Pt-derived compounds with a low IC_50_ value indicates that compounds exhibit cytotoxic properties against MDA-MB-231 and MCF-7 cancer cells. Also, synthesized platinum complexes displayed potent anticancer activity at low concentrations for MCF-7 cells, resulting in cell death. Pt-derived compounds emerged as more potent and selective anticancer drugs compared to the standard drugs cisPt and bical. Therefore, we screened all mono- and bisplatinum complexes for cell cycle analysis. Our results have shown that compounds L_1a_Pt-m, L_1a_Pt-b, and L_1c_Pt-b regulate the cell cycle and increase the G1 population. Furthermore, apoptosis assay revealed that the compounds successfully induce cellular apoptosis in MDA-MB-231 and MCF-7 cancer cells.

## Supplementary Information



## Figures and Tables

**Table 1:** Cytotoxic effect of compounds against CCD-1079Sk, MCF-7, and MDA-MB-231 cell lines.

Code	CCD-1079Sk [IC_50_ (μM)]	MCF-7 [IC_50_ (μM)]	MDA-MB-231 [IC_50_ (μM)]
**L** **_1a_** **Pt-m**	59.52 ± 5.34	8.97 ± 5.28	9.63 ± 7.71
**L** **_1b_** **Pt-m**	54.64 ± 6.25	6.91 ± 3.16	8.64 ± 7.71
**L** **_1c_** **Pt-m**	45.25 ± 5.55	5.56 ± 2.39	25.02 ± 6.61
**L** **_1a_** **Pt-b**	47.77 ± 6.42	5.58 ± 2.29	5.26 ± 5.53
**L** **_1b_** **Pt-b**	67.89 ± 5.11	9.57 ± 5.41	9.73 ± 7.28
**L** **_1c_** **Pt-b**	73.88 ± 4.96	5.90 ± 2.04	6.94 ± 5.57
**CisPt**	27.89 ± 7.24	3.98 ± 1.44	9.16 ± 7.74
**Bical**	9.16 ± 3.18	3.78 ± 1.85	8.34 ± 6.39

**Table 2:** The cell % of apoptotic or necrotic stages in CCD-1079Sk, MDA-MB-231, and MCF-7 cells treated with compounds.

Code	CCD-1079Sk		MCF-7		MDA-MB-231	
	Apoptotic	Necrotic	Apoptotic	Necrotic	Apoptotic	Necrotic
**L** **_1a_** **Pt-m**	2.65 ± 0.11	2.50 ± < 0.001	1.80 ± < 0.001	5.45 ± 1.06	9.35 ± 0.46	0.65 ± 0.21
**L** **_1b_** **Pt-m**	1.90 ± < 0.001	0.40 ± < 0.001	3.65 ± 0.18	0.95 ± 0.21	5.95 ± 0.25	0.65 ± 0.07
**L** **_1c_** **Pt-m**	3.45 ± 0.03	3.25 ± 0.07	5.95 ± 0.46	3.40 ± 0.71	6.20 ± 0.14	0.55 ± 0.07
**L** **_1a_** **Pt-b**	3.65 ± 0.03	4.15 ± 0.21	4.65 ± 0.81	0.95 ± 0.35	7.65 ± 0.25	1.15 ± 0.07
**L** **_1b_** **Pt-b**	1.90 ± 0.07	1.25 ± 0.07	2.95 ± 0.11	2.20 ± 0.14	7.45 ± 0.39	2.65 ± 0.07
**L** **_1c_** **Pt-b**	1.85 ± 0.03	1.80 ± 0.14	2.05 ± 0.03	5.35 ± 1.34	8.00 ± 0.21	3.20 ± 0.14
**CisPt**	5.00 ± 0.07	3.90 ± 0.14	3.45 ± 0.03	1.10 ± 0.14	14.95 ± 0.74	1.55 ± 0.21
**Bical**	5.10 ± 0.21	4.00 ± < 0.001	2.05 ± 0.03	6.35 ± 1.20	6.70 ± 0.28	1.95 ± 0.21
