# Supplementary material for: New imidazolidindionedioximes and their Pt(II) complexes: synthesis and investigation of their antitumoral activities on breast cancer cells
Source: Turk J Chem. 2024 Jan 22;48(4):582–96. doi: 10.55730/1300-0527.3681 (PMC11407372; doi:10.55730/1300-0527.3681)
Supplement: Supplementary file 1 [file TJCHEM-2023-00036-SupplementaryInformation.pdf]

Supplementary information

Contents

1. X-ray data collection, structure resolution and model refinement of 2a

Figure S1. ORTEP plot of 2a, with the atom-numbering scheme. Displacement ellipsoids drawn at the 50% probability level. All H atoms have been omitted. 3
Figure S2. A) The pleated form of the layers, viewed in the direction of propagation of the pleats. B) The N—H···π and C—H···π interactions indicated by dashed lines (symmetry code: –x, –y, –z). 4

Table S. Crystal data and refinement parameters for 2a 4

2. Supplementary figures for synthetic parts

Figure S3. ^{1}H NMR spectrum of N,N'-diphenylmethanediamine (2a) 5
Figure S4. ^{13}C NMR spectrum of N,N'-diphenylmethanediamine (2a) 5
Figure S5. FT-IR spectrum of N,N'-diphenylmethanediamine (2a) 6
Figure S6. MS spectrum of N,N'-diphenylmethanediamine (2a) 6
Figure S7. ^{1}H NMR spectrum of N,N'-bis(4-fluorophenyl)methanediamine (2b) 7
Figure S8. ^{13}C NMR spectrum of N,N'-bis(4-fluorophenyl)methanediamine (2b) 7
Figure S9. FT-IR spectrum of N,N'-bis(4-fluorophenyl)methanediamine (2b) 8
Figure S10. MS spectrum of N,N'-bis(4-fluorophenyl)methanediamine (2b) 8
Figure S11. ^{1}H NMR spectrum of N,N'-bis(4-fluorophenyl)methanediamine (2b) in DMSO-d_6 after 2 h 9
Figure S12. ^{1}H NMR spectrum of N,N'-bis(4-fluorophenyl)methanediamine (2b) in $CDCl_3$ after 30 min 9
Figure S13. FT-IR spectrum of N,N'-bis(4-fluorophenyl)methanediamine (2b) after cyclization 10
Figure S14. ^{1}H NMR spectrum of N,N'-bis(2,4-difluorophenyl)methanediamine (2c) 10
Figure S15. ^{13}C NMR spectrum of N,N'-bis(2,4-difluorophenyl)methanediamine (2c) 11
Figure S16. FT-IR spectrum of N,N'-bis(2,4-difluorophenyl)methanediamine (2c) 11
Figure S17. MS spectrum of N,N'-bis(2,4-difluorophenyl)methanediamine (2c) 12
Figure S18. ^{1}H NMR spectrum of (4Z,5E)-1,3-diphenylimidazolidine-4,5-dionedioxime (L_{1a}) 12
Figure S19. ^{13}C NMR spectrum of (4Z,5E)-1,3-diphenylimidazolidine-4,5-dionedioxime (L_{1a}) 13
Figure S20. FT-IR spectrum of (4Z,5E)-1,3-diphenylimidazolidine-4,5-dionedioxime (L_{1a}) 13
Figure S21. MS spectrum of (4Z,5E)-1,3-diphenylimidazolidine-4,5-dionedioxime (L_{1a}) 14
Figure S22. ^{1}H NMR spectrum of (4Z,5E)-1,3-bis(4-fluorophenyl)imidazolidine-4,5-dionedioxime (L_{1b}) 14
Figure S23. ^{13}C NMR spectrum of (4Z,5E)-1,3-bis(4-fluorophenyl)imidazolidine-4,5-dionedioxime (L_{1b}) 15
Figure S24. FT-IR spectrum of (4Z,5E)-1,3-bis(4-fluorophenyl)imidazolidine-4,5-dionedioxime (L_{1b}) 15
Figure S25. MS spectrum of (4Z,5E)-1,3-bis(4-fluorophenyl)imidazolidine-4,5-dionedioxime (L_{1b}) 16
Figure S26. ^{1}H NMR spectrum of (4Z,5E)-1,3-bis(2,4-difluorophenyl)imidazolidine-4,5-dionedioxime (L_{1c}) 16
Figure S27. ^{13}C NMR spectrum of (4Z,5E)-1,3-bis(2,4-difluorophenyl)imidazolidine-4,5-dionedioxime (L_{1c}) 17
Figure S28. FT-IR spectrum of (4Z,5E)-1,3-bis(2,4-difluorophenyl)imidazolidine-4,5-dionedioxime (L_{1c}) 17
Figure S29. MS spectrum of (4Z,5E)-1,3-bis(2,4-difluorophenyl)imidazolidine-4,5-dionedioxime (L_{1c}) 18
Figure S30. FT-IR spectrum of (4Z,5E)-1,3-diphenylimidazolidine-4,5-dionedioxime mono-Pt(II) complex (L_{1a}Pt-m) 18
Figure S31. MS spectrum of (4Z,5E)-1,3-diphenylimidazolidine-4,5-dionedioxime mono-Pt(II) complex (L_{1a}Pt-m) 19

Figure S32. FT-IR spectrum of (4Z,5E)-1,3-bis(4-fluorophenyl)imidazolidine-4,5-dionedioxime mono-Pt(II) complex (L_{1b}Pt-m) 19
Figure S33. MS spectrum of (4Z,5E)-1,3-bis(4-fluorophenyl)imidazolidine-4,5-dionedioxime mono-Pt(II) complex (L_{1b}Pt-m) 20
Figure S34. FT-IR spectrum of (4Z,5E)-1,3-bis(2,4-difluorophenyl)imidazolidine-4,5-dionedioxime mono-Pt(II) complex (L_{1c}Pt-m) 20
Figure S35. MS spectrum of (4Z,5E)-1,3-bis(2,4-difluorophenyl)imidazolidine-4,5-dionedioxime mono-Pt(II) complex (L_{1c}Pt-m) 21
Figure S36. FT-IR spectrum of (4Z,5E)-1,3-diphenylimidazolidine-4,5-dionedioxime bis-Pt(II) complex (L_{1a}Pt-b) 21
Figure S37. MS spectrum of (4Z,5E)-1,3-diphenylimidazolidine-4,5-dionedioxime bis-Pt(II) complex (L_{1a}Pt-b) 22
Figure S38. FT-IR spectrum of (4Z,5E)-1,3-bis(4-fluorophenyl)imidazolidine-4,5-dionedioxime bis-Pt(II) complex (L_{1b}Pt-b) 22
Figure S39. MS spectrum of (4Z,5E)-1,3-bis(4-fluorophenyl)imidazolidine-4,5-dionedioxime bis-Pt(II) complex (L_{1b}Pt-b) 23
Figure S40. FT-IR spectrum of (4Z,5E)-1,3-bis(2,4-difluorophenyl)imidazolidine-4,5-dionedioxime bis-Pt(II) complex (L_{1c}Pt-b) 23
Figure S41. MS spectrum of (4Z,5E)-1,3-bis(2,4-difluorophenyl)imidazolidine-4,5-dionedioxime bis-Pt(II) complex (L_{1c}Pt-b) 24

3. Materials and methods for biological studies

4. Supplementary figures for biological studies

Figure S42. Cell cycle analysis of compounds treated A) CCD-1079Sk, B) MCF-7, and C) MDA-MB-231 cells. 26
Figure S43. Apoptosis assay results of compounds treated A) CCD-1079Sk, B) MCF-7, and C) MDA-MB-231 cells. 28

1. X-ray data collection, structure resolution and model refinement of 2a

The crystal data were obtained with Bruker APEX II QUAZAR three-circle diffractometer. Indexing was performed using APEX2 [S1]. Data integration and reduction were carried out with SAINT [S2]. Absorption correction was performed by multi-scan method implemented in SADABS [S3]. The structure was solved using SHELXT [S4] and then refined by full-matrix least-squares refinements on F^2 using the SHELXL [S4] in OLEX2 [S5]. All nonhydrogen atoms were refined anisotropically using all reflections with $I > 2\sigma(I)$. Aromatic and aliphatic C-bound H atoms were positioned geometrically and refined using a riding mode. Crystallographic data and refinement details of the data collection for **2a** are given in **Table S** Mercury was used for visualization of the cif file [S6]. Additional crystallographic data with CCDC reference number 2306536 have been deposited within the Cambridge Crystallographic Data Center via the following link: www.ccdc.cam.ac.uk/deposit

N,N'-Diphenylmethanediamine **2a**, $C_{13}H_{14}N_2$, forms triclinic crystals. A view of the compound **2a** is shown on **Figure S1**. In the crystal structure, the intermolecular interactions are primarily of N—H···π and C—H···π character, without any contribution from N—H···N hydrogen bonding. Taken together, N—H···π and C—H···π interactions form crystal package of the structure (**Figure S2**).

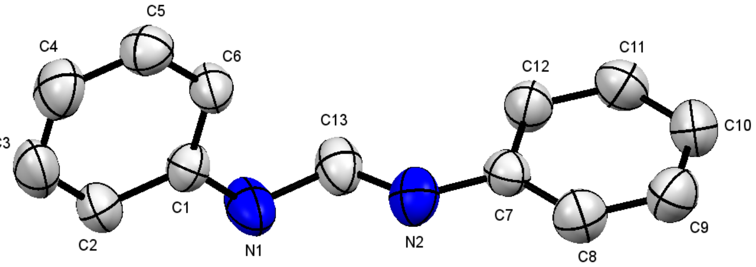

Figure S1. ORTEP plot of 2a, with the atom-numbering scheme. Displacement ellipsoids drawn at the 50% probability level. All H atoms have been omitted.

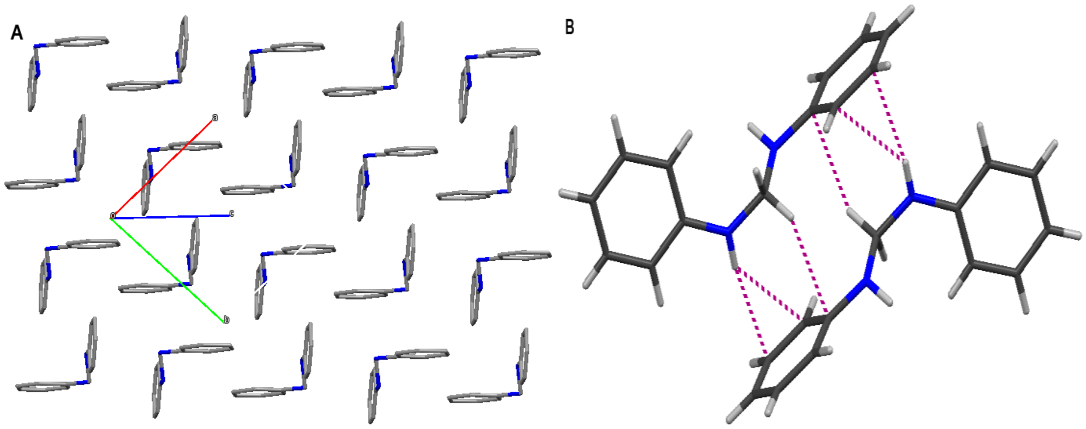

Figure S2. A) The pleated form of the layers, viewed in the direction of propagation of the pleats. B) The N—H···π and C—H···π interactions indicated by dashed lines (symmetry code: –x, –y, –z).

Table S. Crystal data and refinement parameters for 2a.

**Empirical formula**	$0.5(C_{6.5}H_7N)$
**Formula weight (g mol^{-1})**	99.13
**Temperature (K)**	296.15
**Crystal system**	Triclinic
**Space group**	*P*-1
a **(Å)**	7.118 (4)
b **(Å)**	7.596 (4)
c **(Å)**	10.967 (6)
**α (°)**	72.24 (4)
**β (°)**	77.59 (4)
**γ (°)**	85.39 (4)
**Crystal size (mm)**	0.244 × 0.12 × 0.047
V **(Å^3)**	551.5(6)
Z	4
**ρ_{calcd} (g cm^{-3})**	1.194
**μ (mm^{-1})**	0.072
***F*(000)**	212.0
**2 θ range for data collection (°)**	3.98 to 49.996
h*/*k*/*l	$-8 \le h \le 8$, $-6 \le k \le 8$, $-13 \le l \le 13$
**Reflections collected**	4175
**Independent reflections**	1849 [Rint = 0.0772, Rsigma = 0.1122]
**Data/restraints/parameters**	1851/0/137
**Goodness-of-fit on F^2**	0.892
**Final *R* indices [$I > 2\sigma(I)$]**	*R1 = 0.0748, wR2 = 0.1759*
R **indices (all data)**	*R1 = 0.1607, wR2 = 0.2143*
**Largest diff. peak and hole (e.Å^{-3})**	0.46/–0.39
**CCDC**	2306536

2. Supplementary figures for synthetic parts

2.1 *N*,*N'*-diphenylmethanediamine (2a)

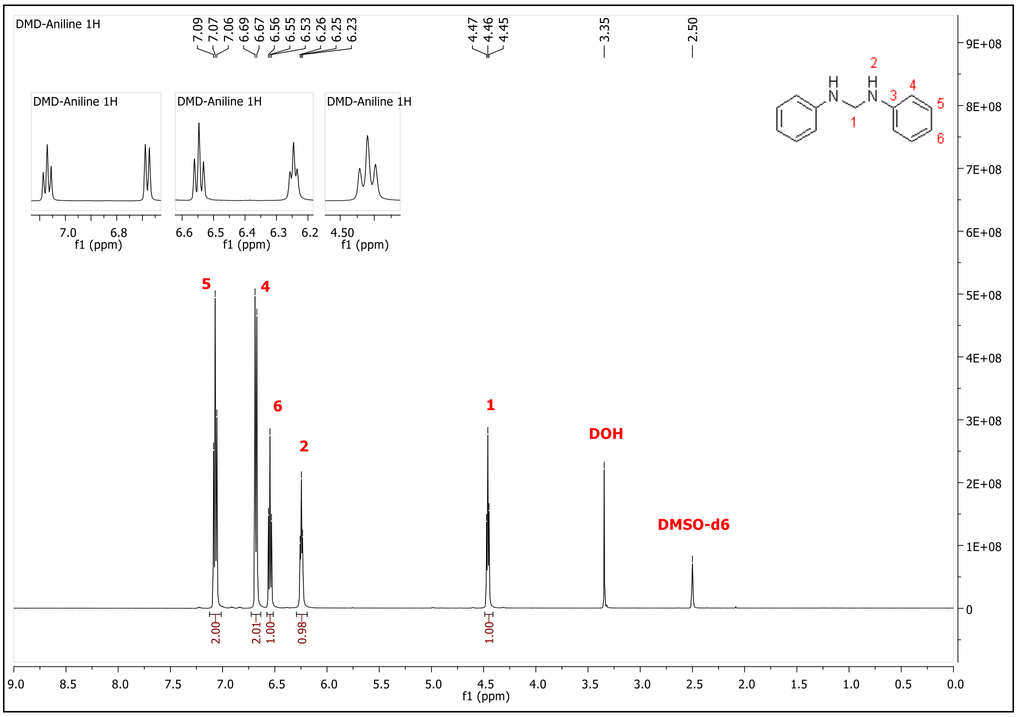

Figure S3. ^{1}H NMR spectrum of *N*,*N'*-diphenylmethanediamine (2a).

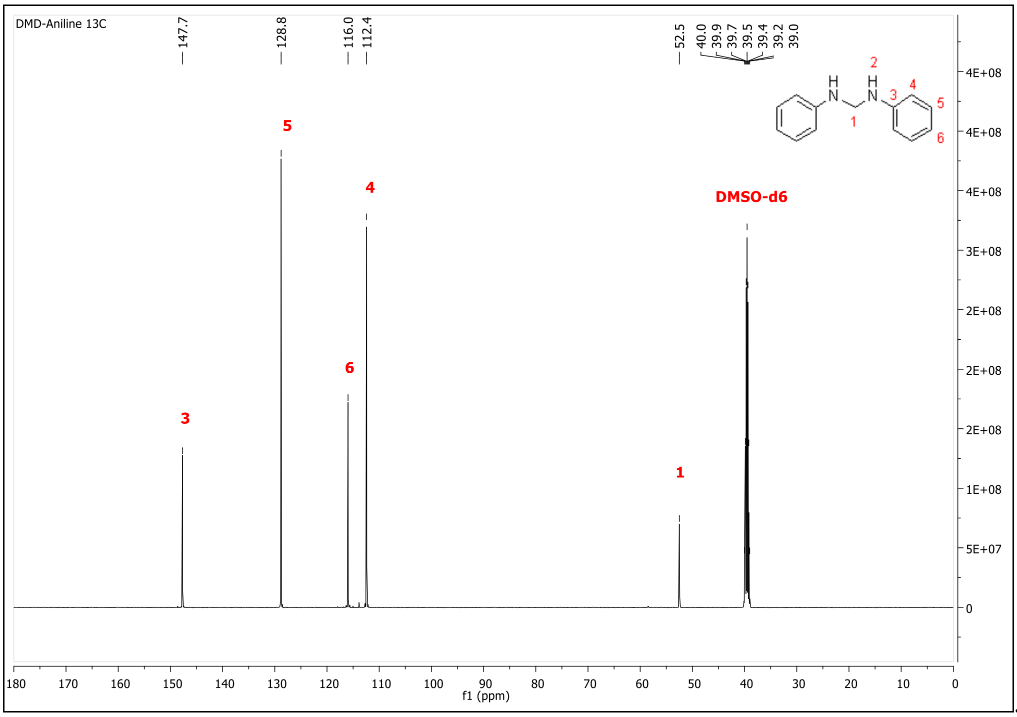

Figure S4. ^{13}C NMR spectrum of *N*,*N'*-diphenylmethanediamine (2a).

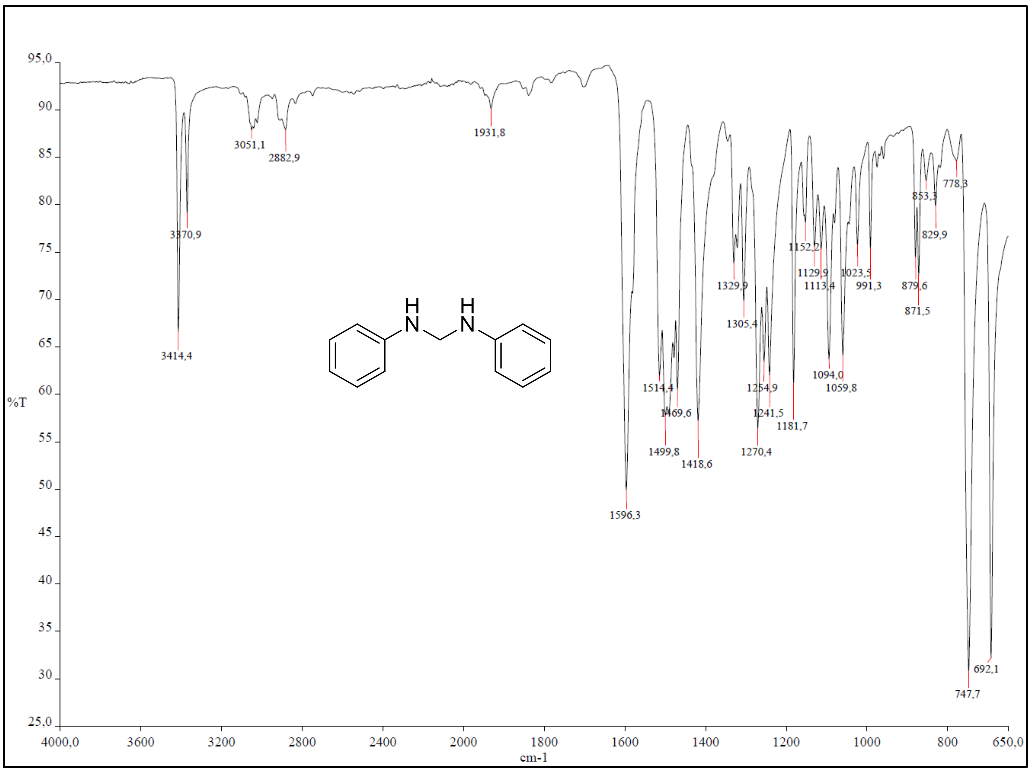

Figure S5. FT-IR spectrum of *N*,*N'*-diphenylmethanediamine (2a).

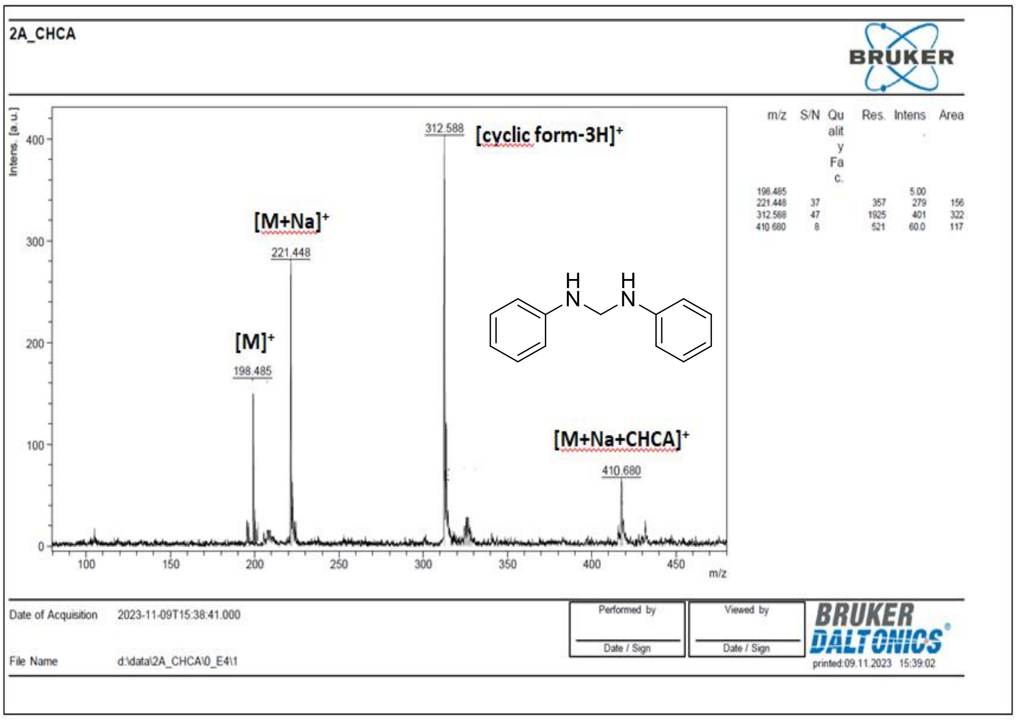

Figure S6. MS spectrum of *N*,*N'*-diphenylmethanediamine (2a).

2.2 *N*,*N'*-bis(4-fluorophenyl)methanediamine

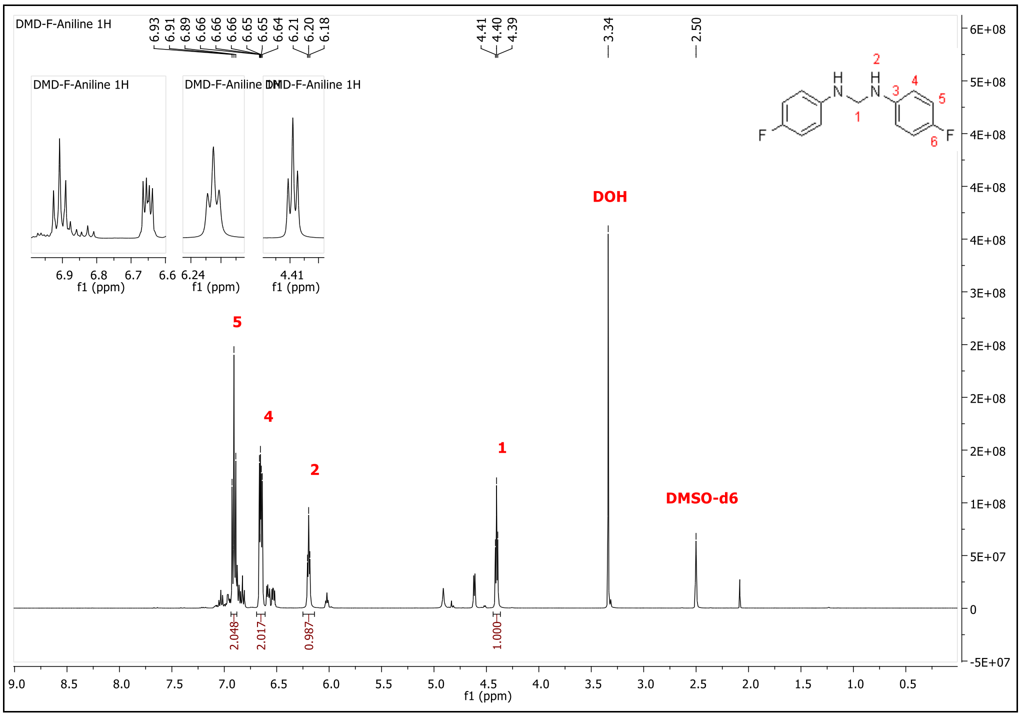

Figure S7. ^{1}H NMR spectrum of *N*,*N'*-bis(4-fluorophenyl)methanediamine (2b).

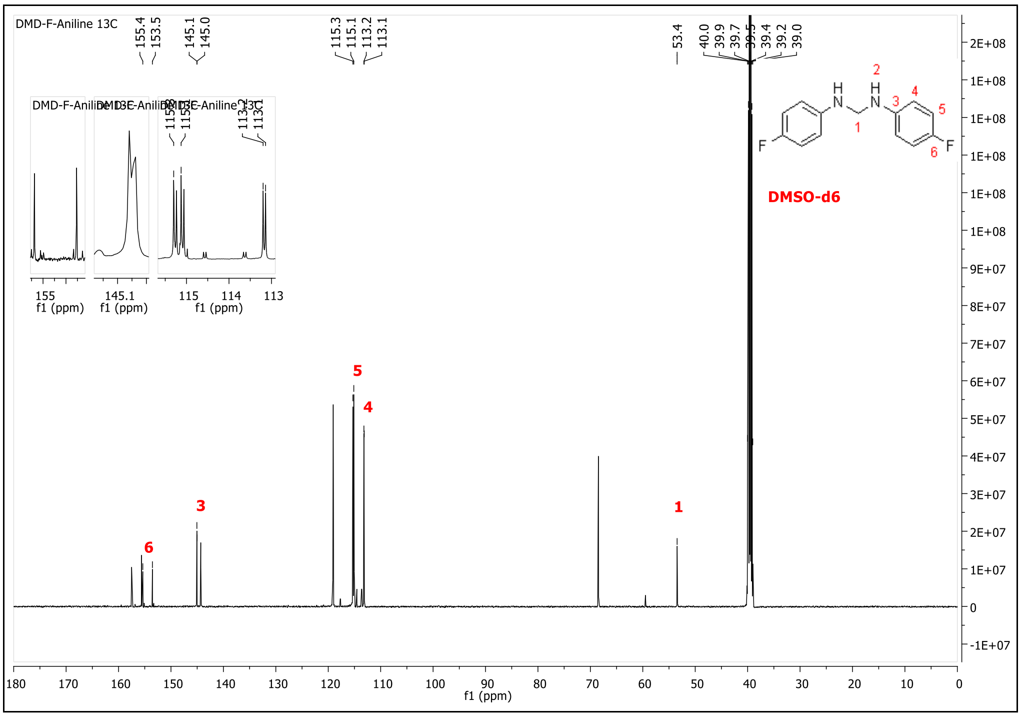

Figure S8. ^{13}C NMR spectrum of *N*,*N'*-bis(4-fluorophenyl)methanediamine (2b).

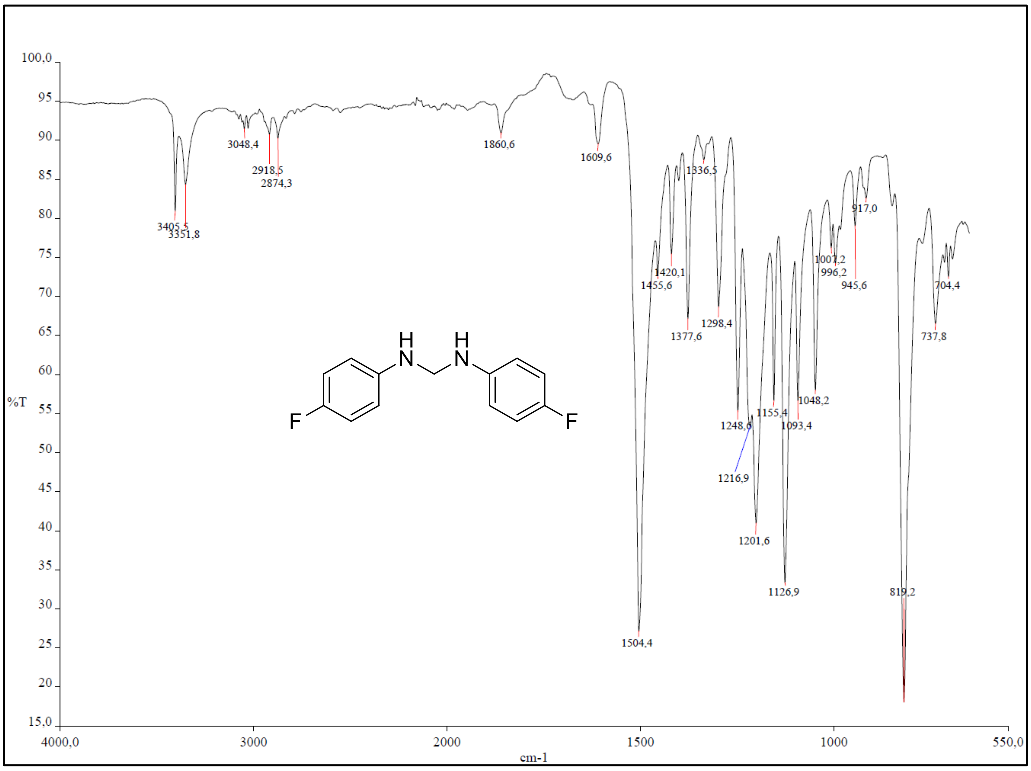

Figure S9. FT-IR spectrum of *N*,*N'*-bis(4-fluorophenyl)methanediamine (2b).

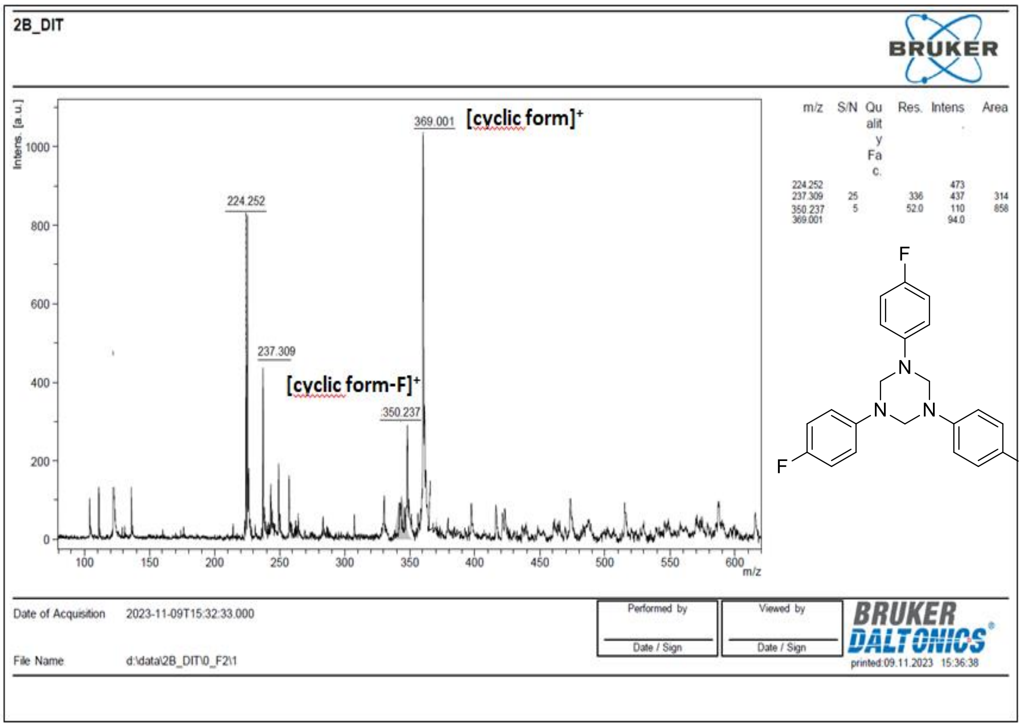

Figure S10. MS spectrum of *N*,*N'*-bis(4-fluorophenyl)methanediamine (2b).

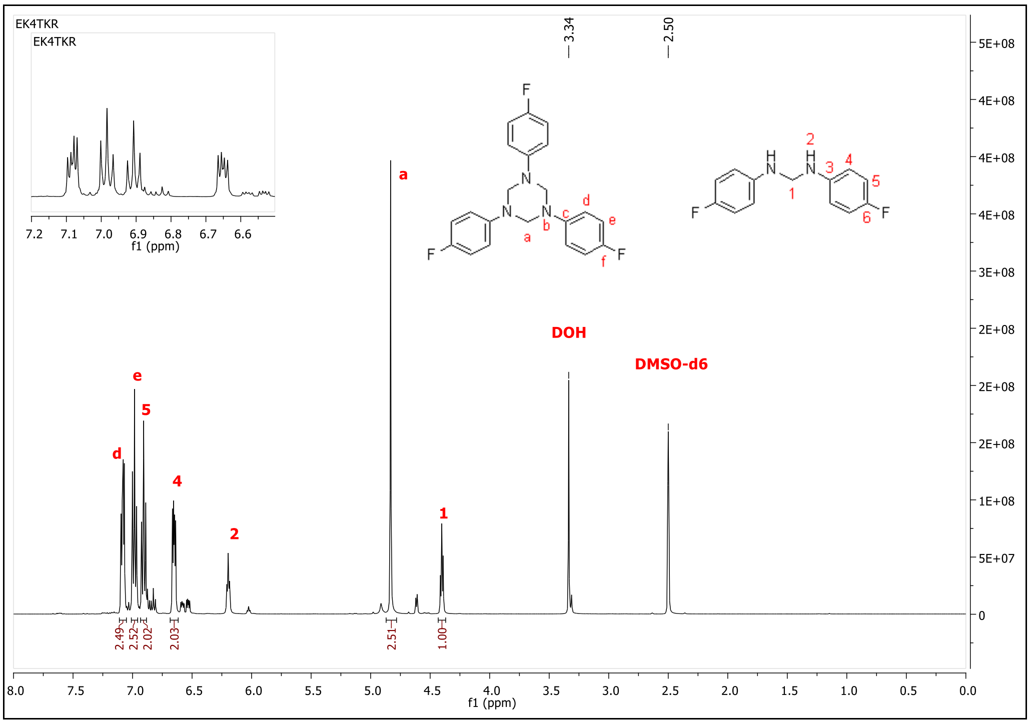

Figure S11. ^{1}H NMR spectrum of *N*,*N'*-bis(4-fluorophenyl)methanediamine (2b) in DMSO-d_6 after 2 h.

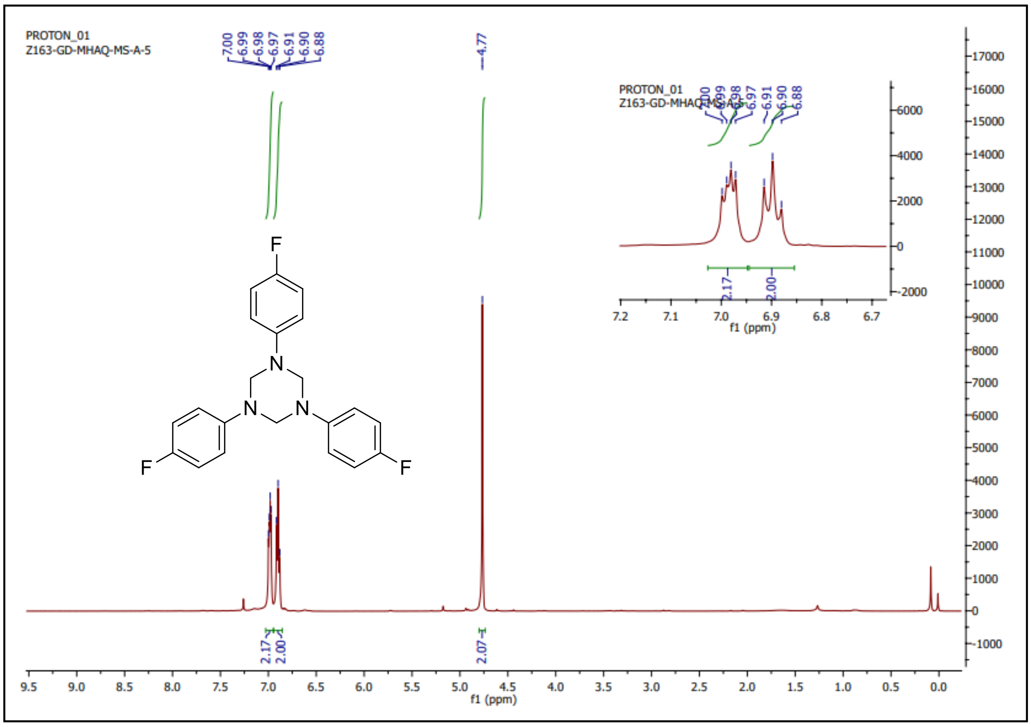

Figure S12. ^{1}H NMR spectrum of *N*,*N'*-bis(4-fluorophenyl)methanediamine (2b) in $CDCl_3$ after 30 min.

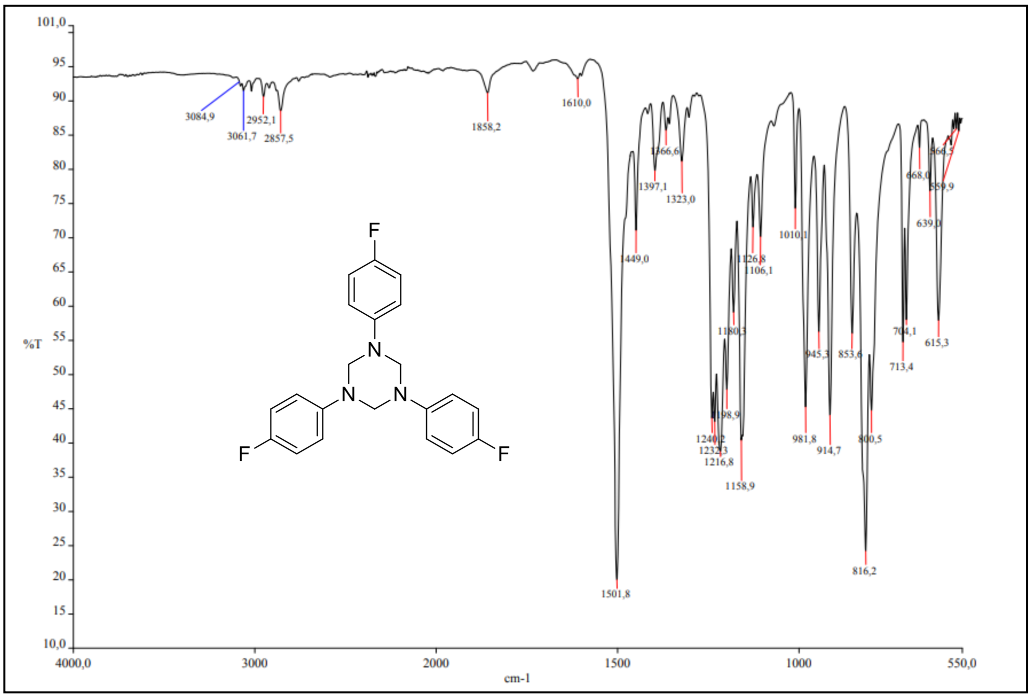

Figure S13. FT-IR Spectrum of *N,N'*-bis(4-fluorophenyl)methanediamine (2b) after cyclization.

2.3 *N,N'*-bis(2,4-difluorophenyl)methanediamine

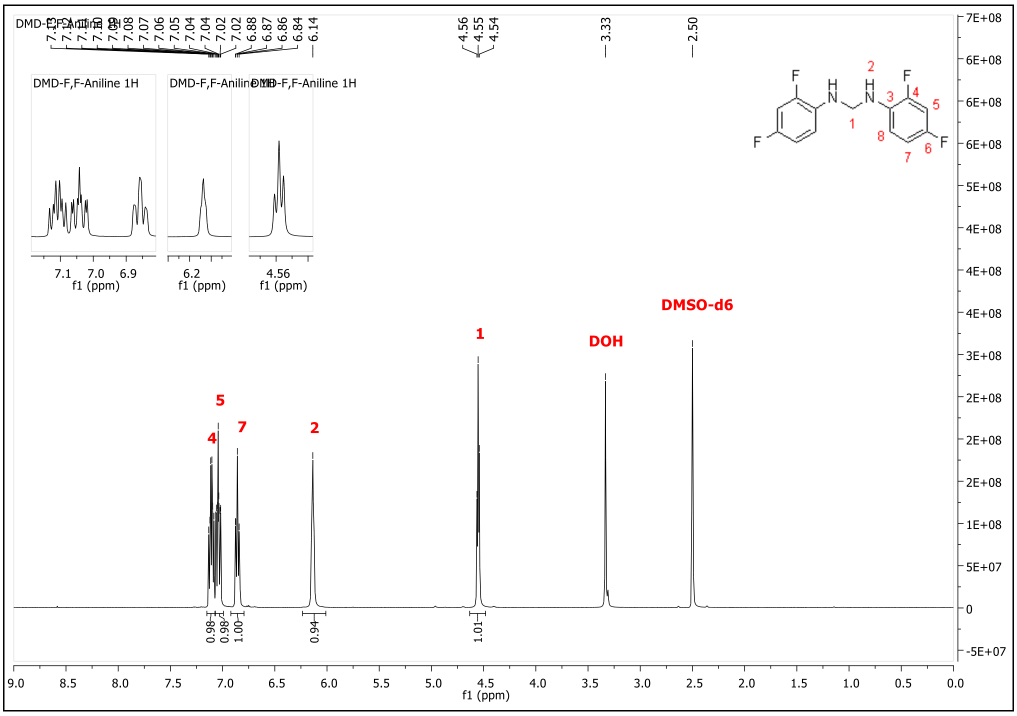

Figure S14. ^{1}H NMR spectrum of *N,N'*-bis(2,4-difluorophenyl)methanediamine (2c).

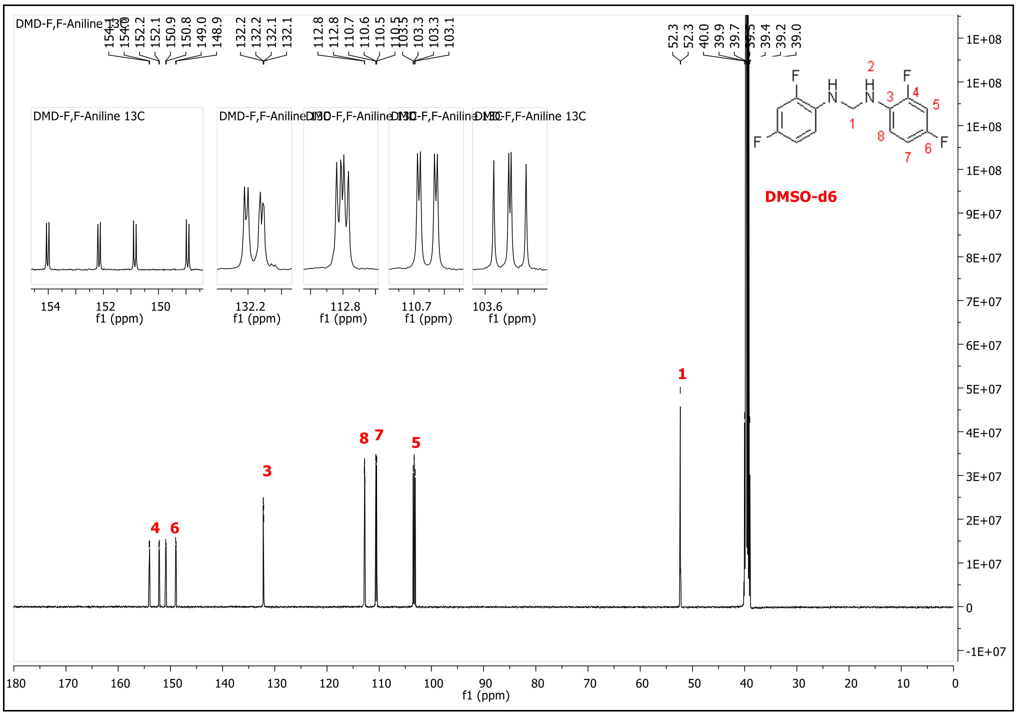

Figure S15. ^{13}C NMR spectrum of *N*,*N'*-bis(2,4-difluorophenyl)methanediamine (2c).

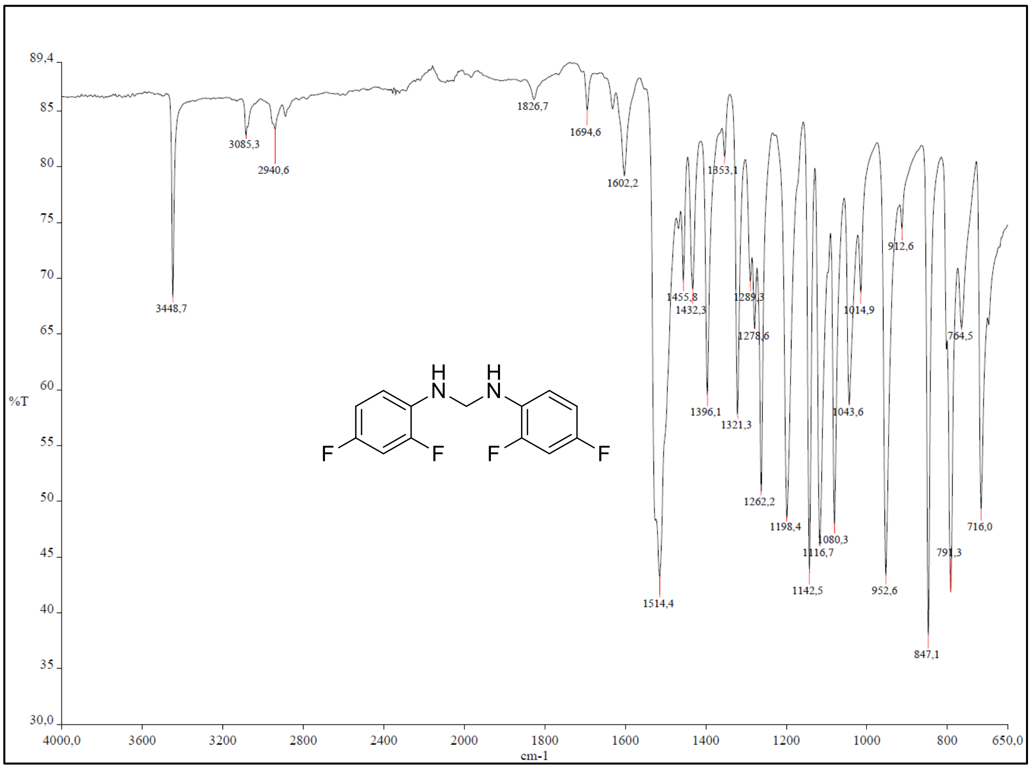

Figure S16. FT-IR spectrum of *N*,*N'*-bis(2,4-difluorophenyl)methanediamine (2c).

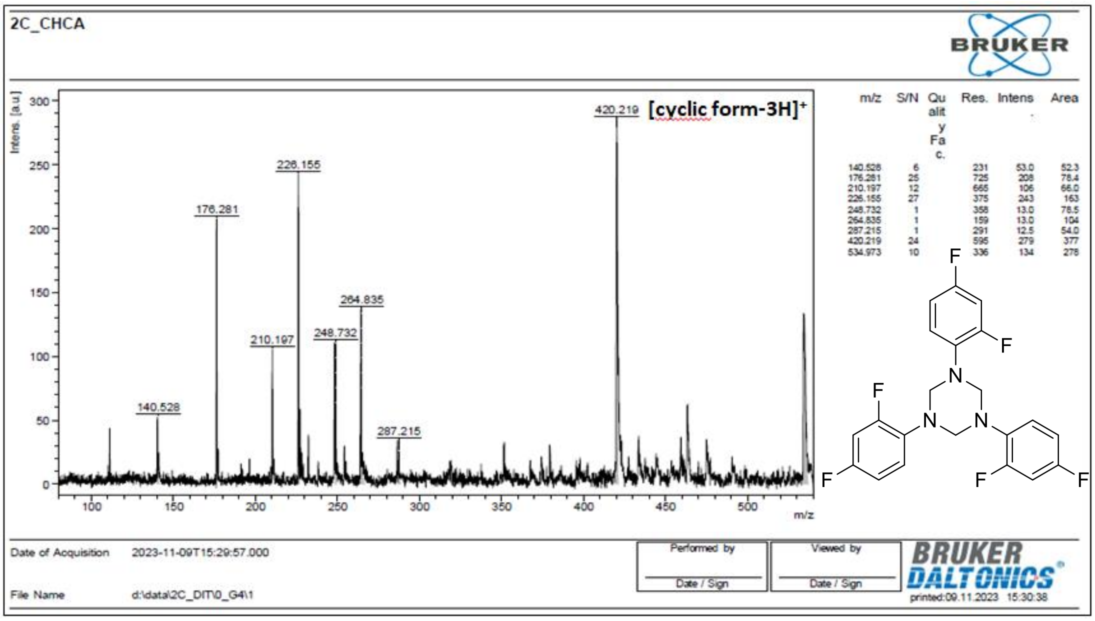

Figure S17. MS spectrum of *N*,*N'*-bis(2,4-difluorophenyl)methanediamine (2c).

2.4 (4Z,5E)-1,3-diphenylimidazolidine-4,5-dionedioxime

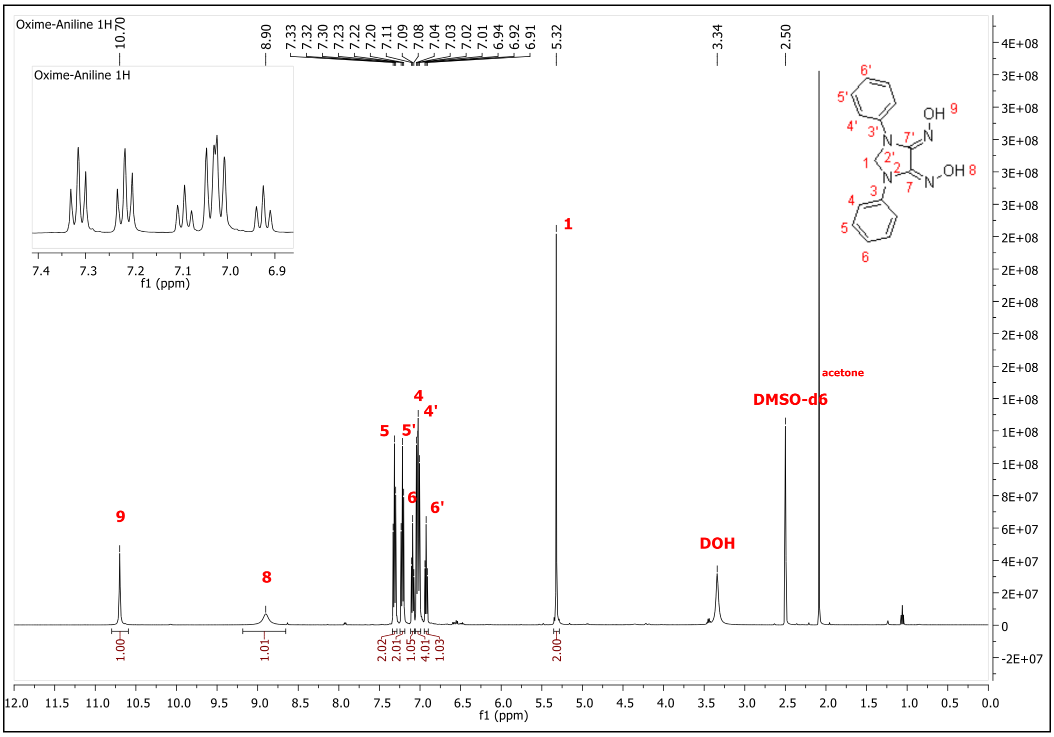

Figure S18. ^{1}H NMR spectrum of (4Z,5E)-1,3-diphenylimidazolidine-4,5-dionedioxime (L_{1a}).

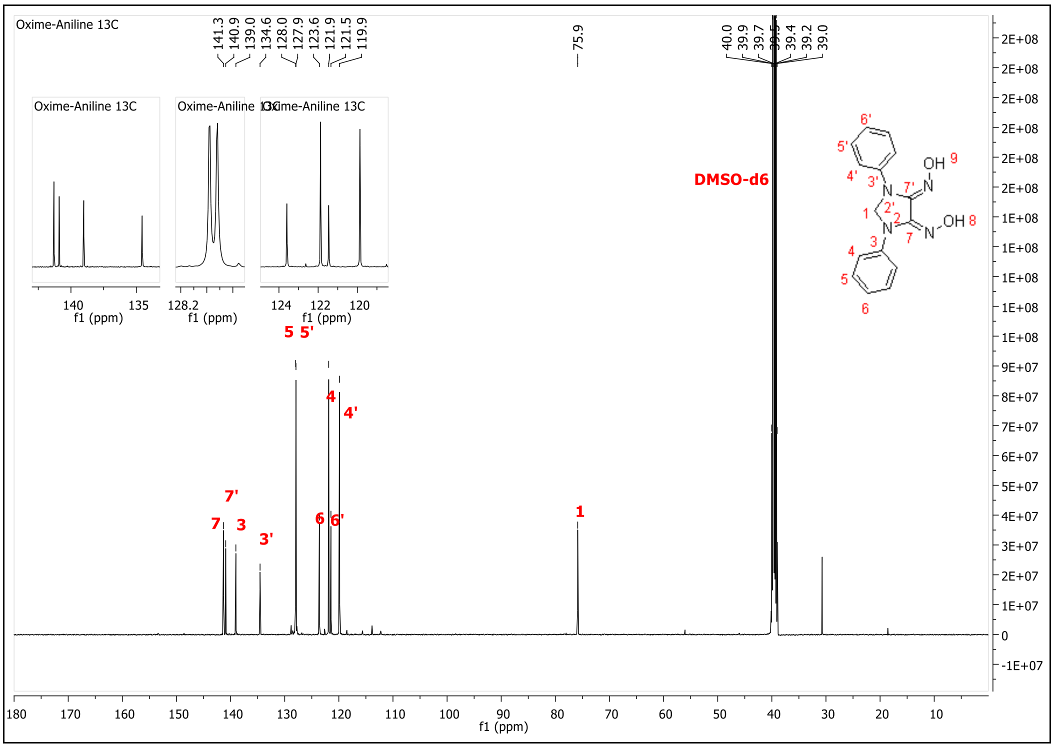

Figure S19. ^{13}C NMR spectrum of (4Z,5E)-1,3-diphenylimidazolidine-4,5-dionedioxime (L_{1a}).

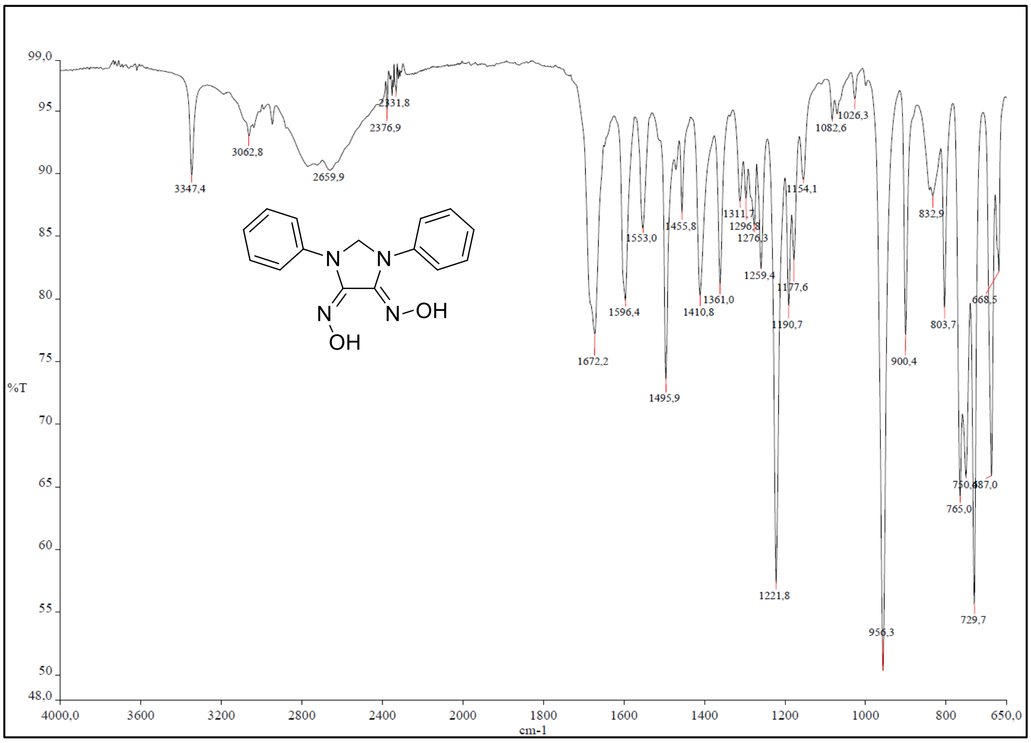

Figure S20. FT-IR spectrum of (4Z,5E)-1,3-diphenylimidazolidine-4,5-dionedioxime (L_{1a}).

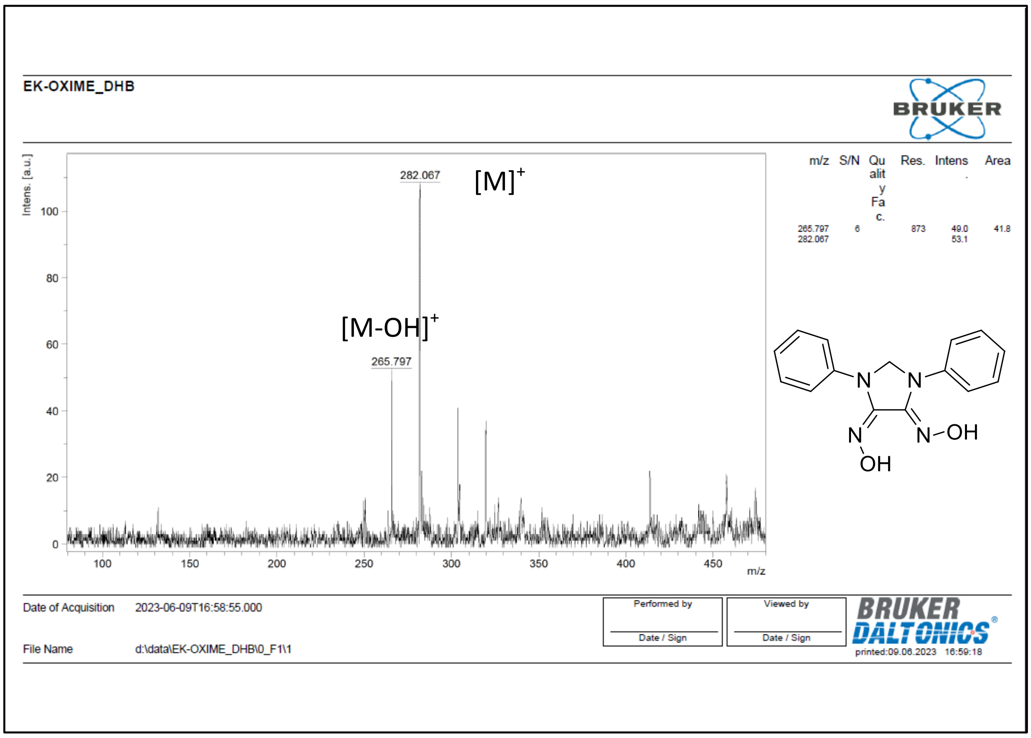

Figure S21. MS spectrum of (4Z,5E)-1,3-diphenylimidazolidine-4,5-dionedioxime (L_{1a}).

2.5 (4Z,5E)-1,3-bis(4-fluorophenyl)imidazolidine-4,5-dionedioxime

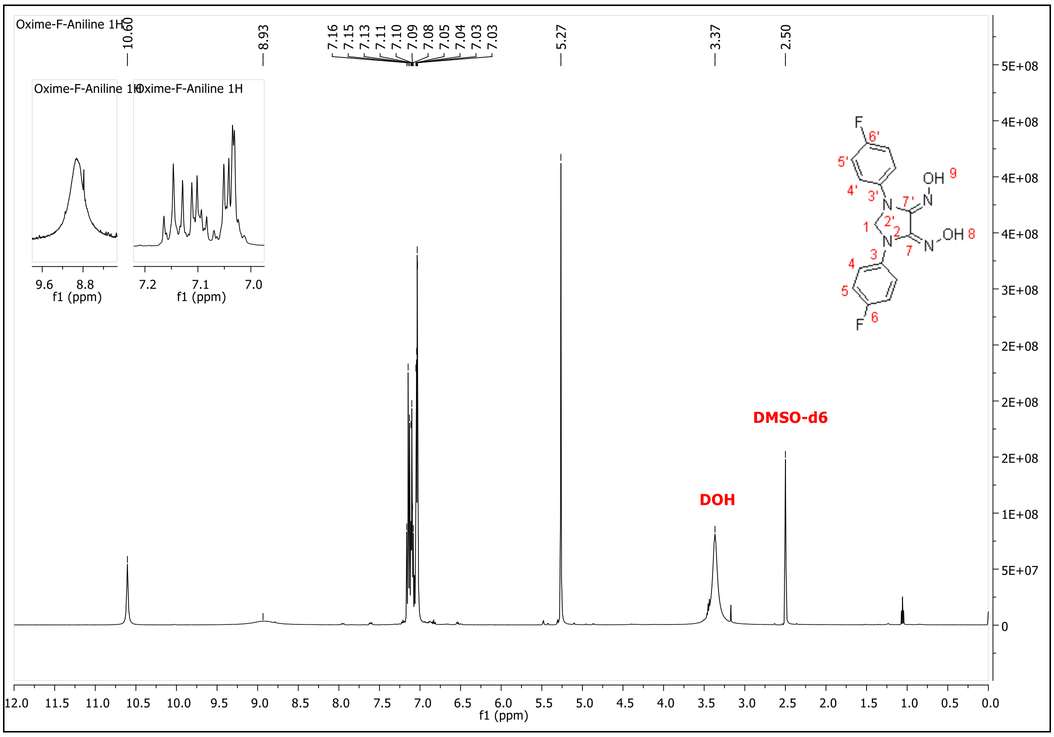

Figure S22. ^{1}H NMR spectrum of (4Z,5E)-1,3-bis(4-fluorophenyl)imidazolidine-4,5-dionedioxime (L_{1b}).

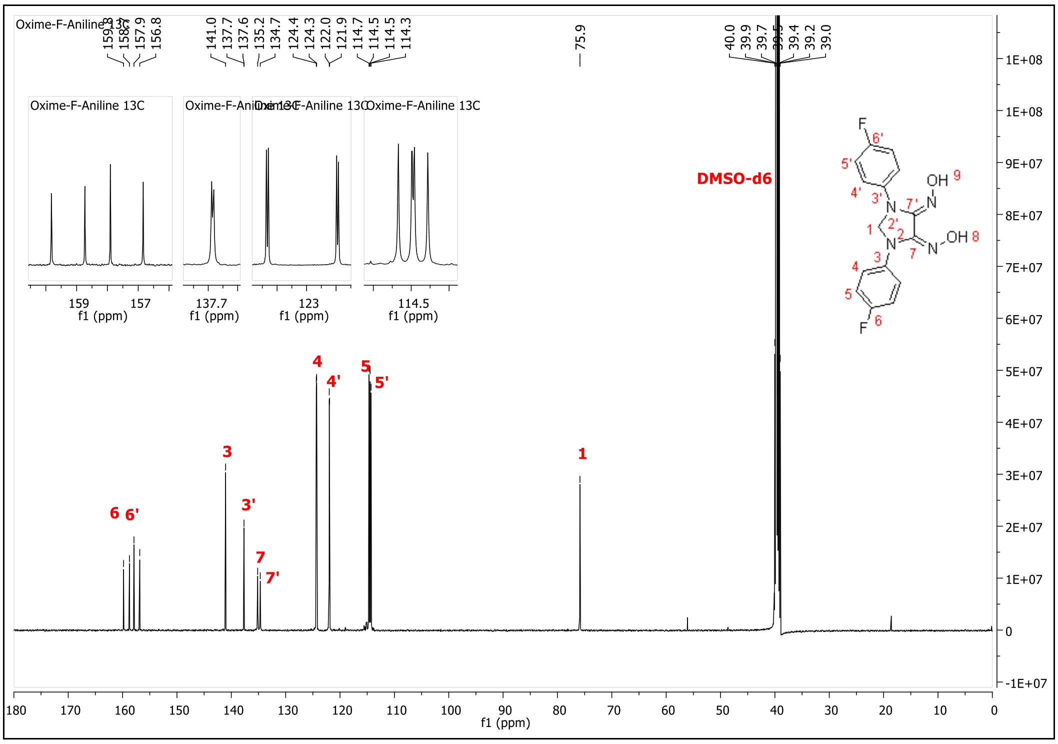

Figure S23. ^{13}C NMR spectrum of (4Z,5E)-1,3-bis(4-fluorophenyl)imidazolidine-4,5-dionedioxime (L_{1b}).

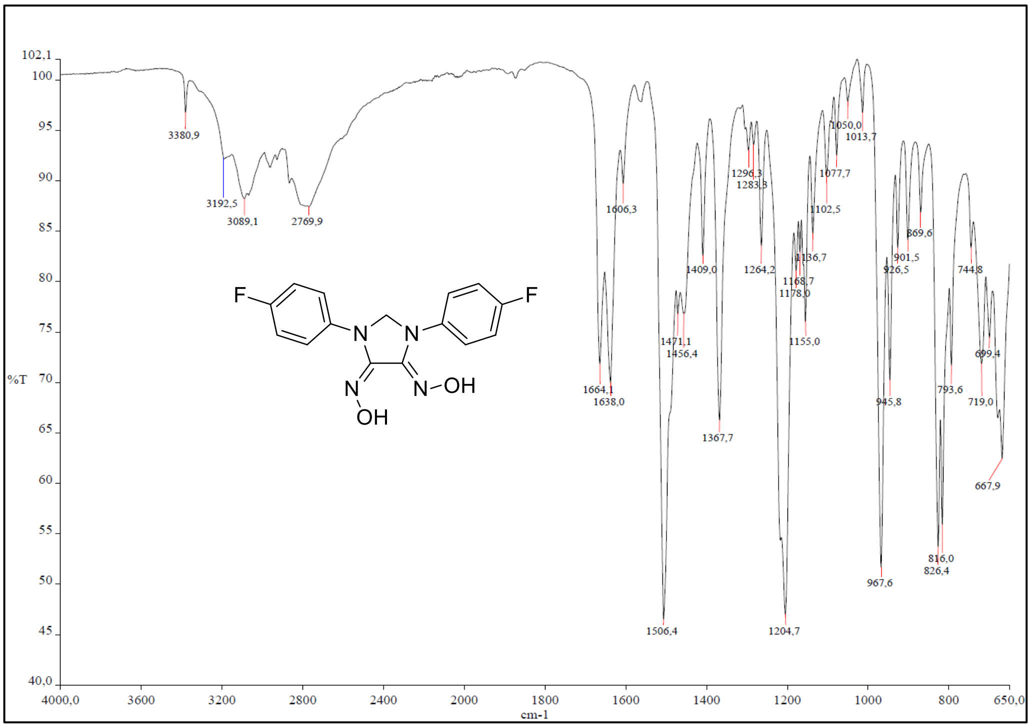

Figure S24. FT-IR spectrum of (4Z,5E)-1,3-bis(4-fluorophenyl)imidazolidine-4,5-dionedioxime (L_{1b}).

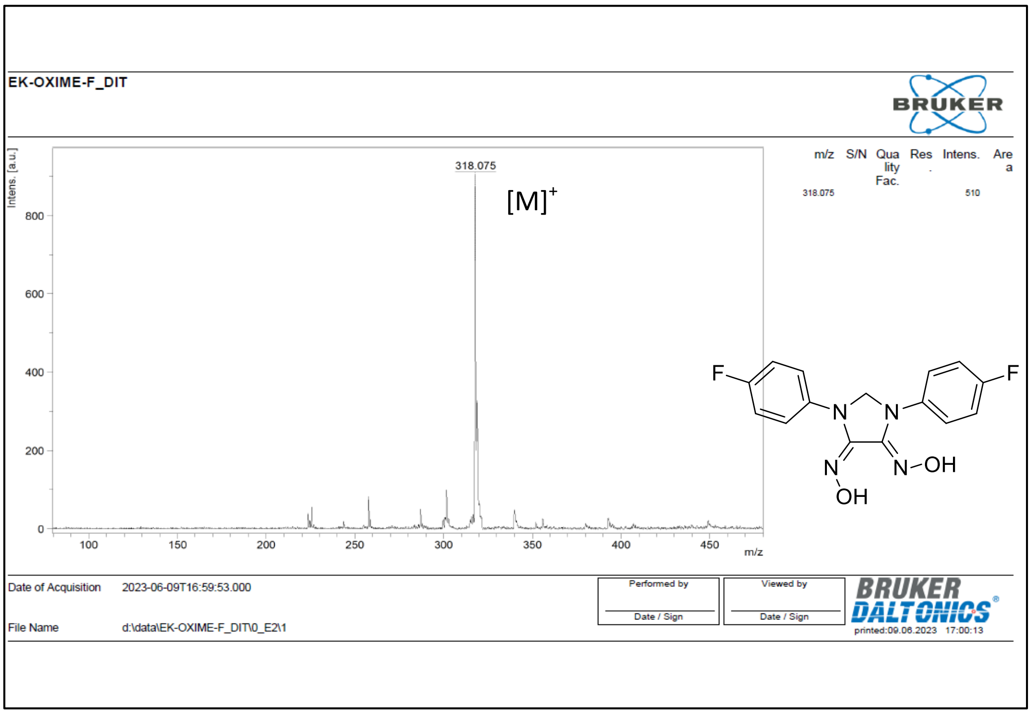

Figure S25. MS spectrum of (4Z,5E)-1,3-bis(4-fluorophenyl)imidazolidine-4,5-dionedioxime (L_{1b}).

2.6 (4Z,5E)-1,3-bis(2,4-difluorophenyl)imidazolidine-4,5-dionedioxime

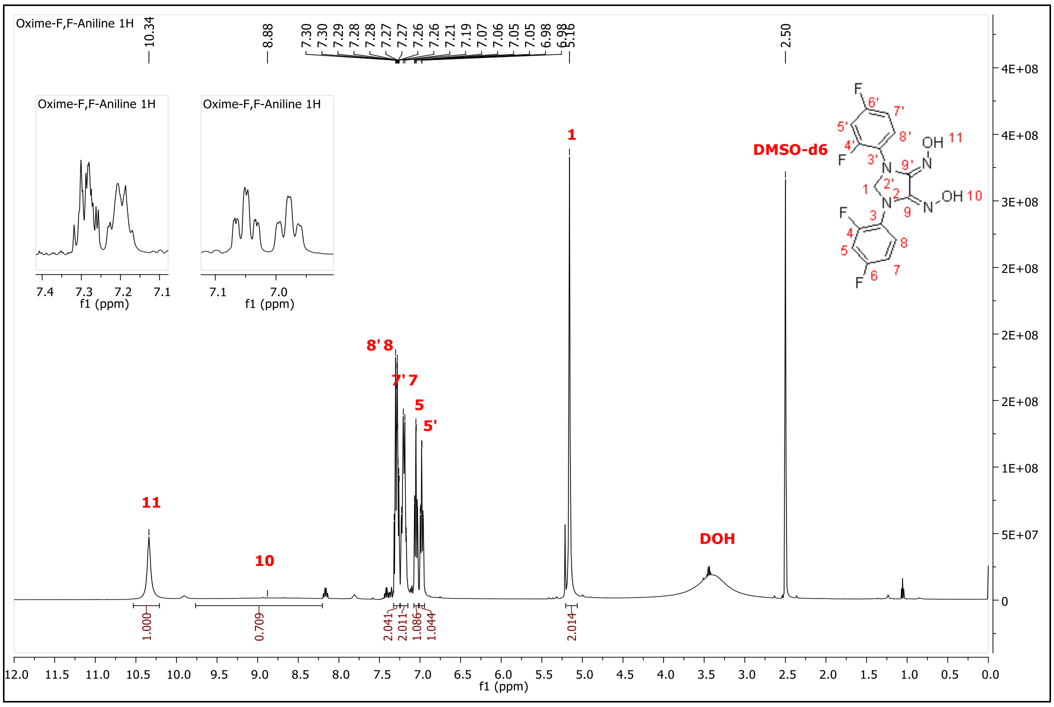

Figure S26. ^{1}H NMR spectrum of (4Z,5E)-1,3-bis(2,4-difluorophenyl)imidazolidine-4,5-dionedioxime (L_{1c}).

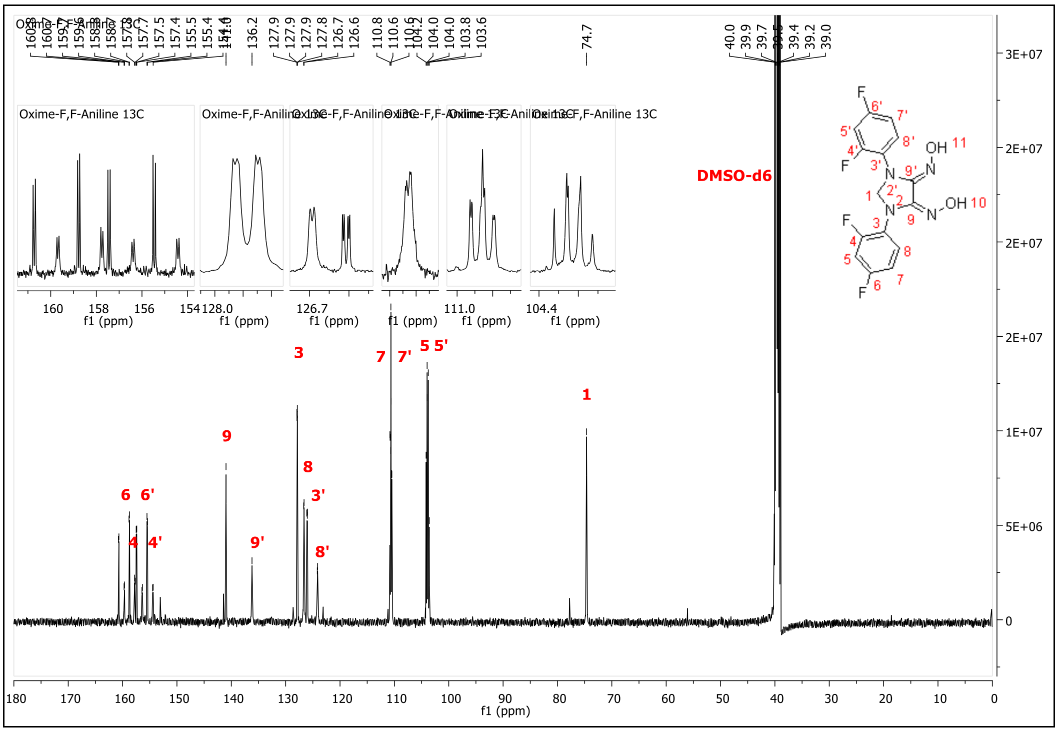

Figure S27. ^{13}C NMR spectrum of (4Z,5E)-1,3-bis(2,4-difluorophenyl)imidazolidine-4,5-dionedioxime (L_{1c}).

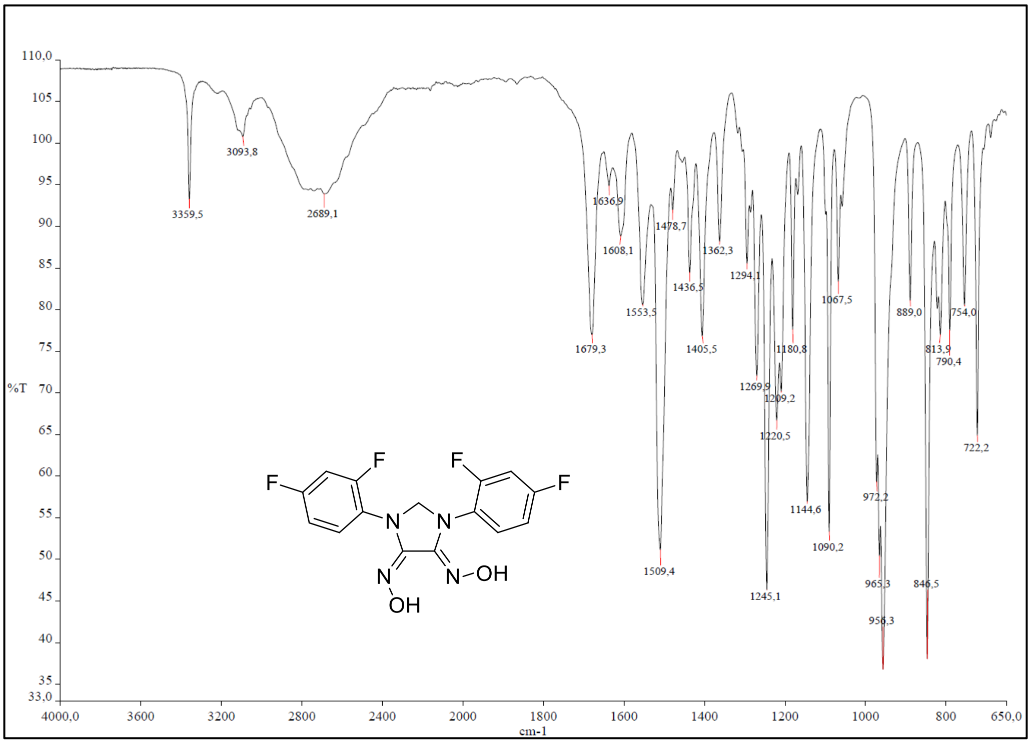

Figure S28. FT-IR spectrum of (4Z,5E)-1,3-bis(2,4-difluorophenyl)imidazolidine-4,5-dionedioxime (L_{1c}).

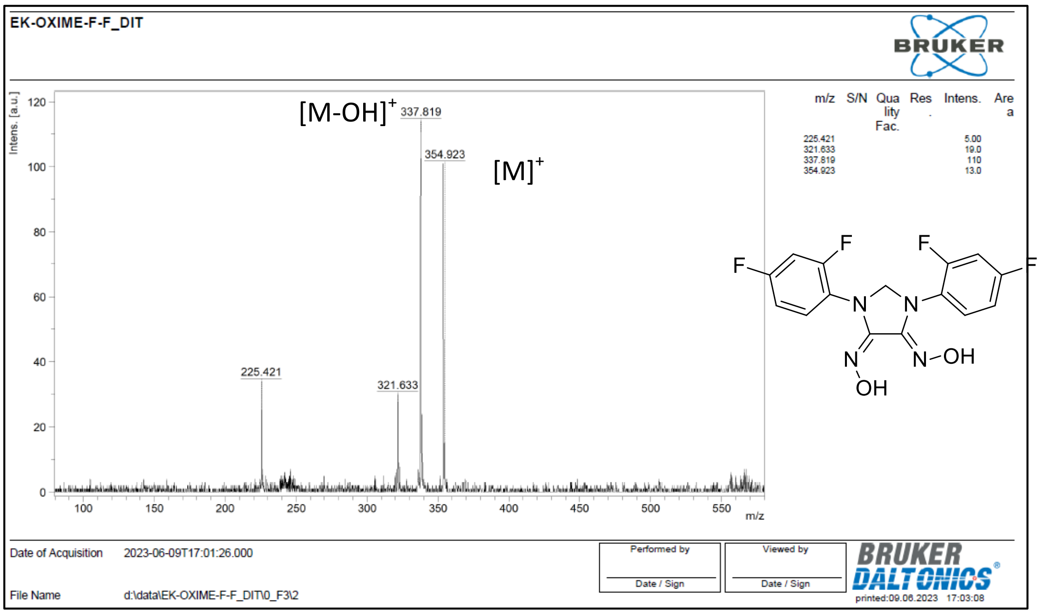

Figure S29. MS spectrum of (4Z,5E)-1,3-bis(2,4-difluorophenyl)imidazolidine-4,5-dionedioxime (L_{1c}).

2.7 Monoplatinum complexes of *vic*-dioximes

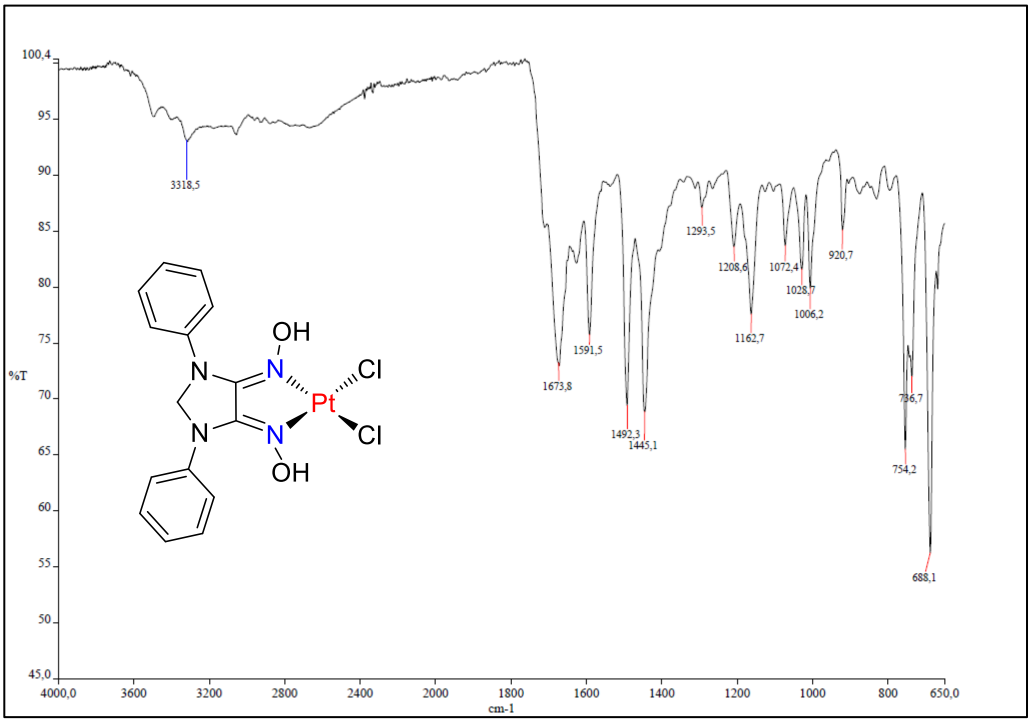

Figure S30. FT-IR spectrum of (4Z,5E)-1,3-diphenylimidazolidine-4,5-dionedioxime mono-Pt(II) complex (L_{1a}Pt-m).

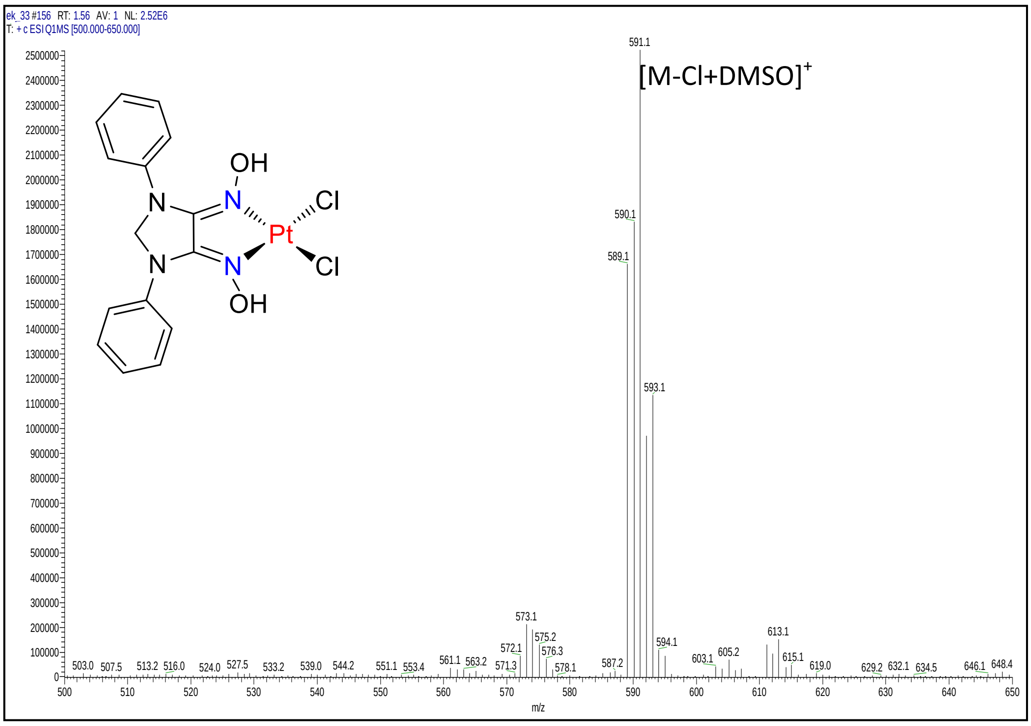

Figure S31. MS spectrum of (4Z,5E)-1,3-diphenylimidazolidine-4,5-dionedioxime mono-Pt(II) complex (L_{1a}Pt-m).

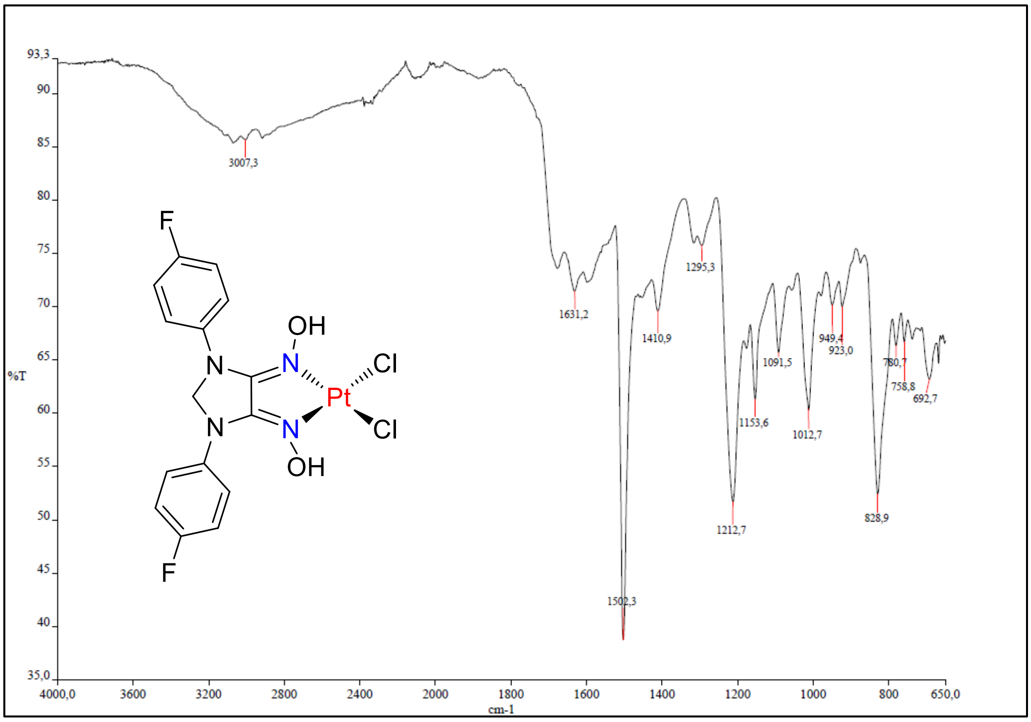

Figure S32. FT-IR spectrum of (4Z,5E)-1,3-bis(4-fluorophenyl)imidazolidine-4,5-dionedioxime mono-Pt(II) complex (L_{1b}Pt-m).

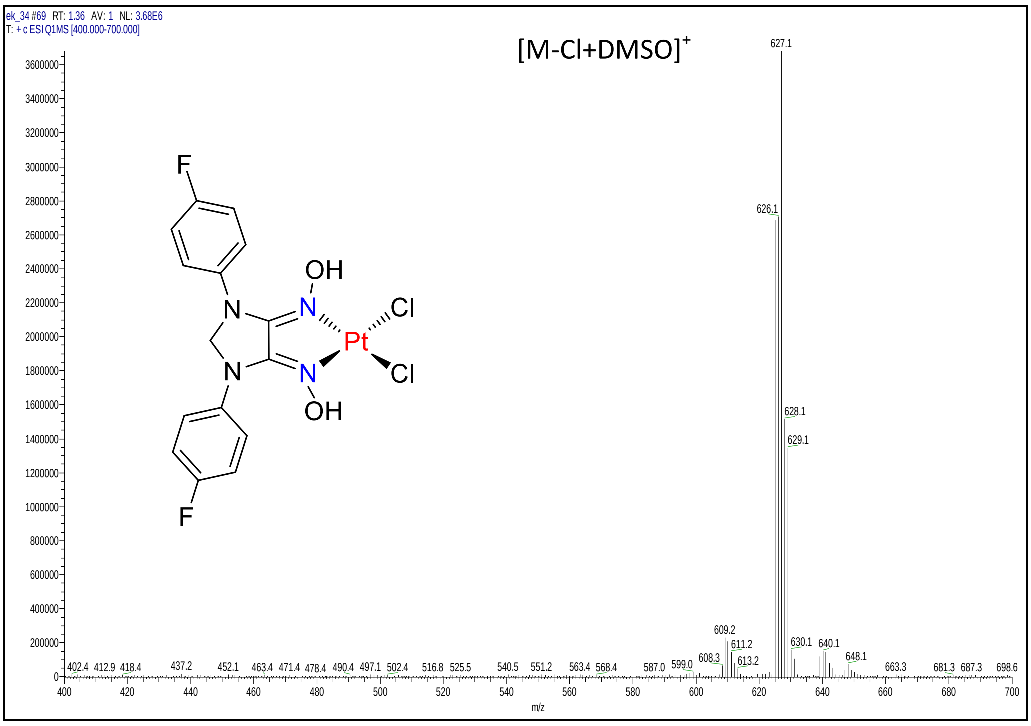

Figure S33. MS spectrum of (4Z,5E)-1,3-bis(4-fluorophenyl)imidazolidine-4,5-dionedioxime mono-Pt(II) complex (L_{1b}Pt-m).

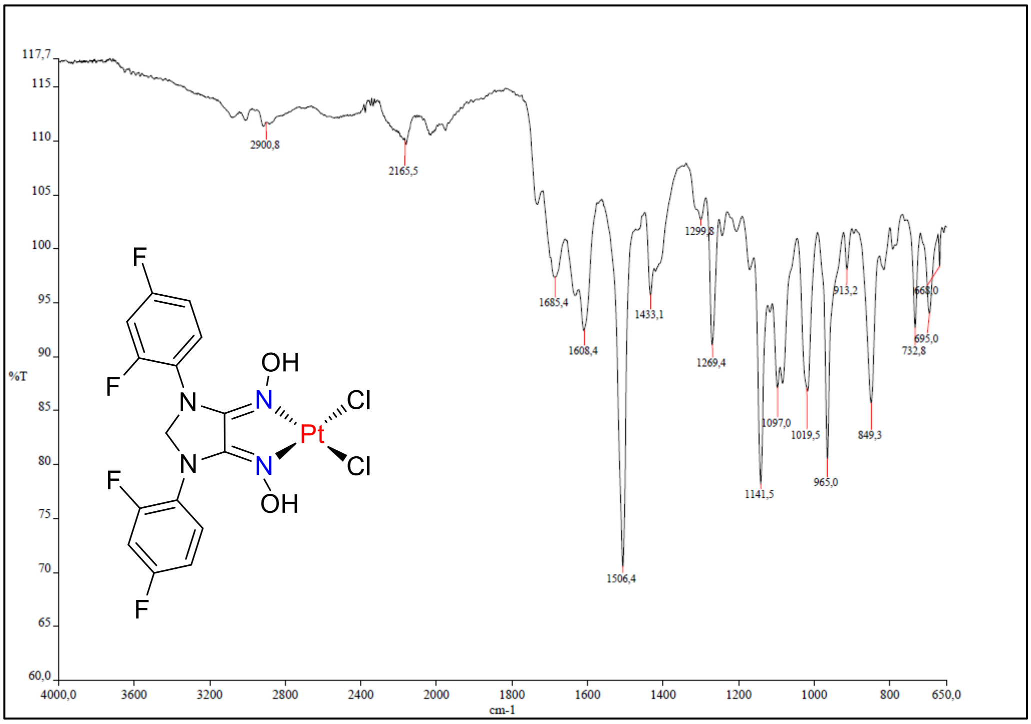

Figure S34. FT-IR spectrum of (4Z,5E)-1,3-bis(2,4-difluorophenyl)imidazolidine-4,5-dionedioxime mono-Pt(II) complex (L_{1c}Pt-m).

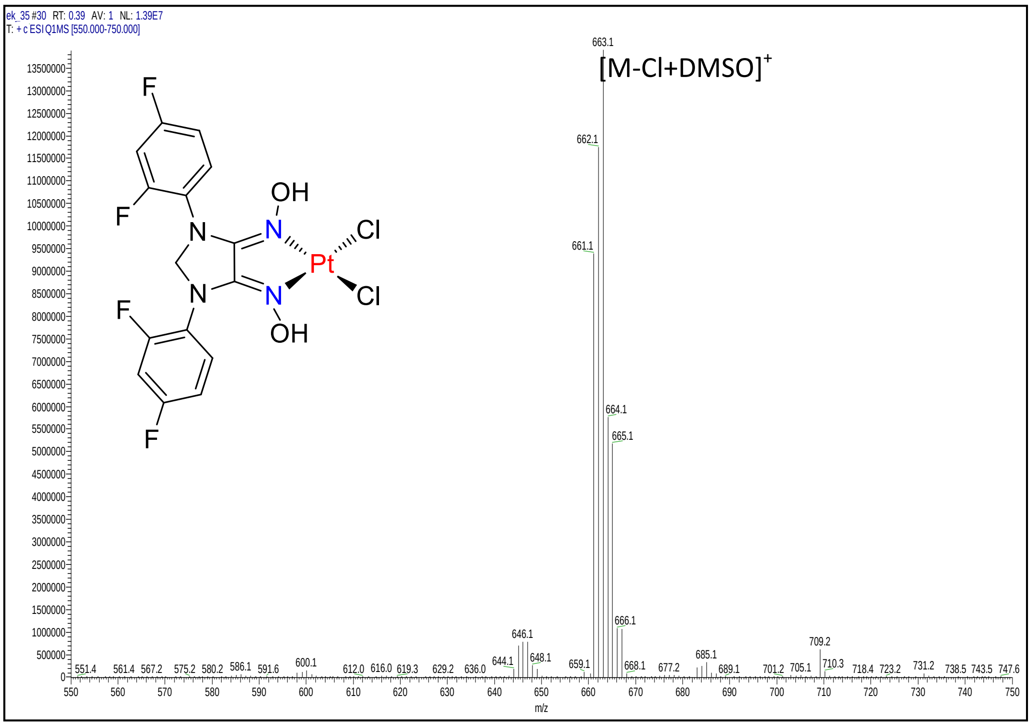

Figure S35. MS spectrum of (4Z,5E)-1,3-bis(2,4-difluorophenyl)imidazolidine-4,5-dionedioxime mono-Pt(II) complex (L_{1c}Pt-m).

2.8 Bisplatinum complexes of *vic*-dioximes

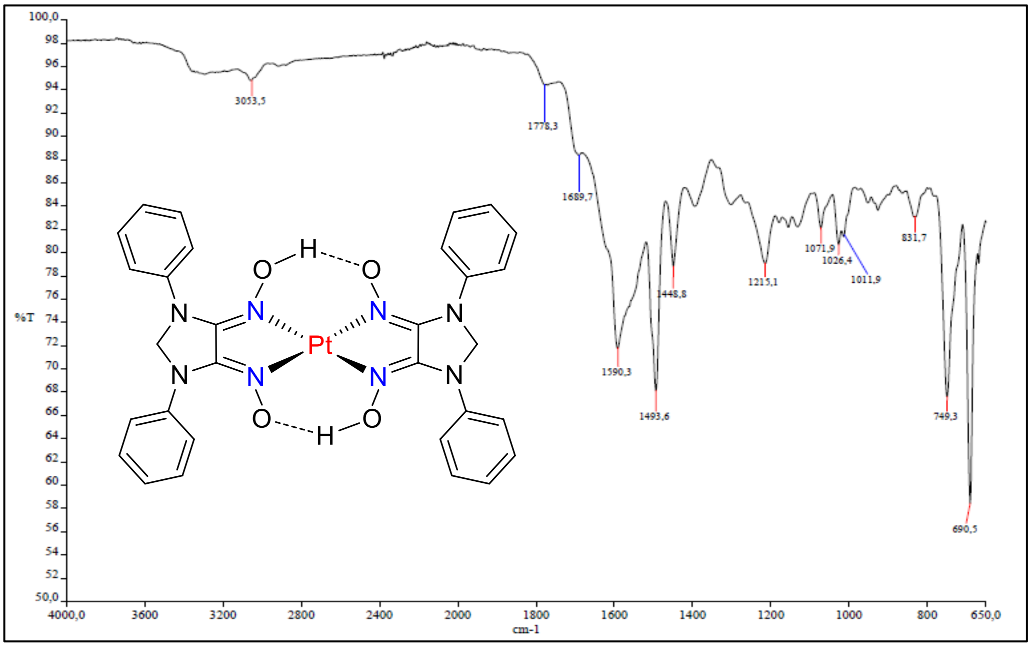

Figure S36. FT-IR spectrum of (4Z,5E)-1,3-diphenylimidazolidine-4,5-dionedioxime bis-Pt(II) complex (L_{1a}Pt-b).

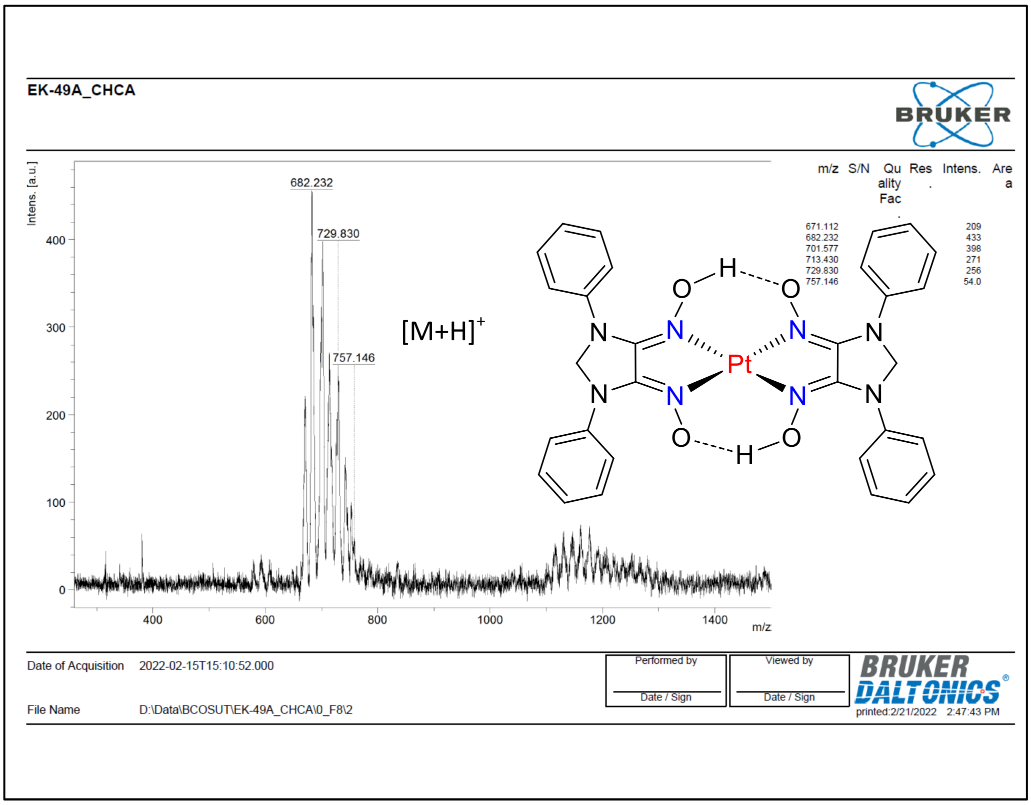

Figure S37. MS spectrum of (4Z,5E)-1,3-diphenylimidazolidine-4,5-dionedioxime bis-Pt(II) complex (L_{1a}Pt-b).

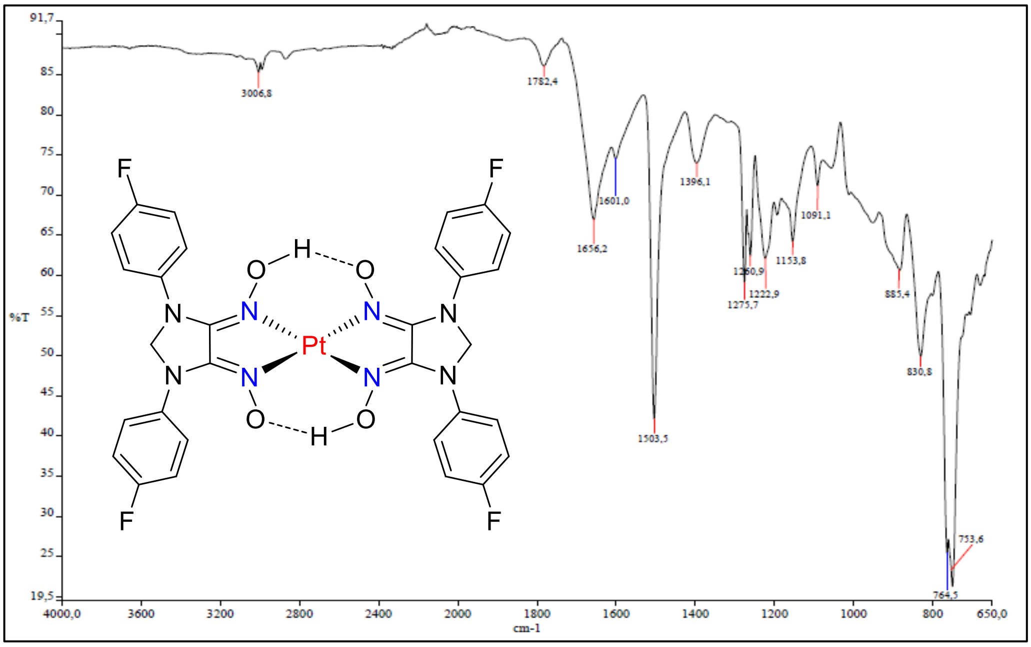

Figure S38. FT-IR spectrum of (4Z,5E)-1,3-bis(4-fluorophenyl)imidazolidine-4,5-dionedioxime bis-Pt(II) complex (L_{1b}Pt-b).

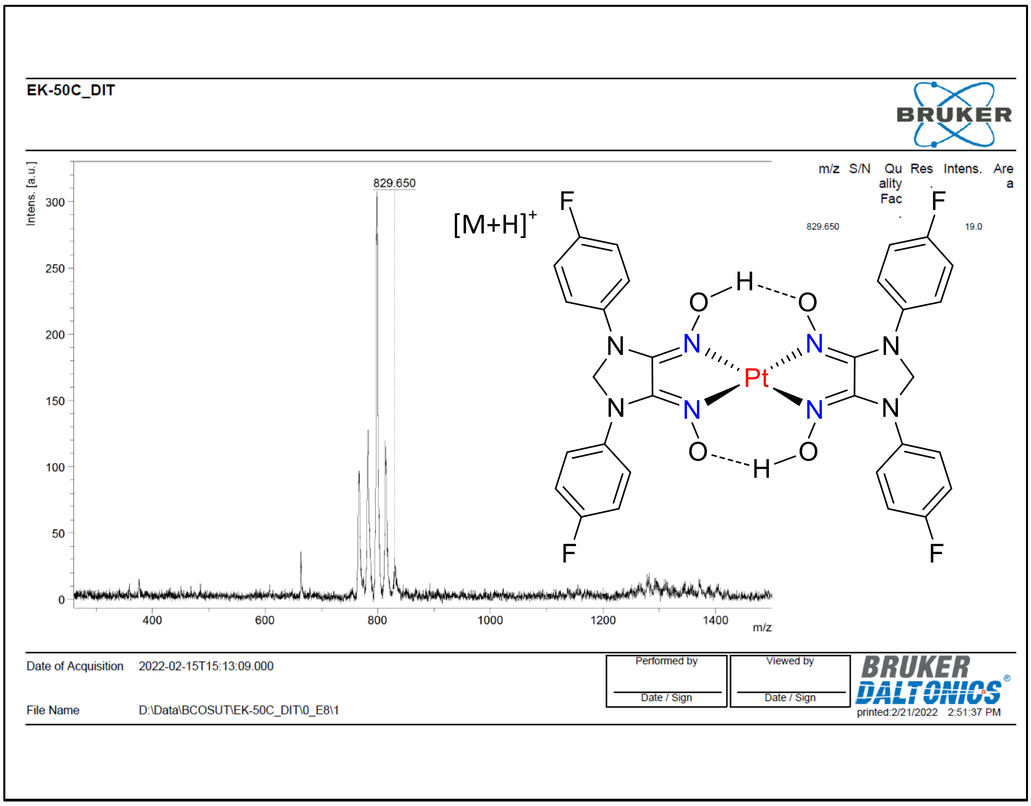

Figure S39. MS spectrum of (4Z,5E)-1,3-bis(4-fluorophenyl)imidazolidine-4,5-dionedioxime bis-Pt(II) complex (L_{1b}Pt-b).

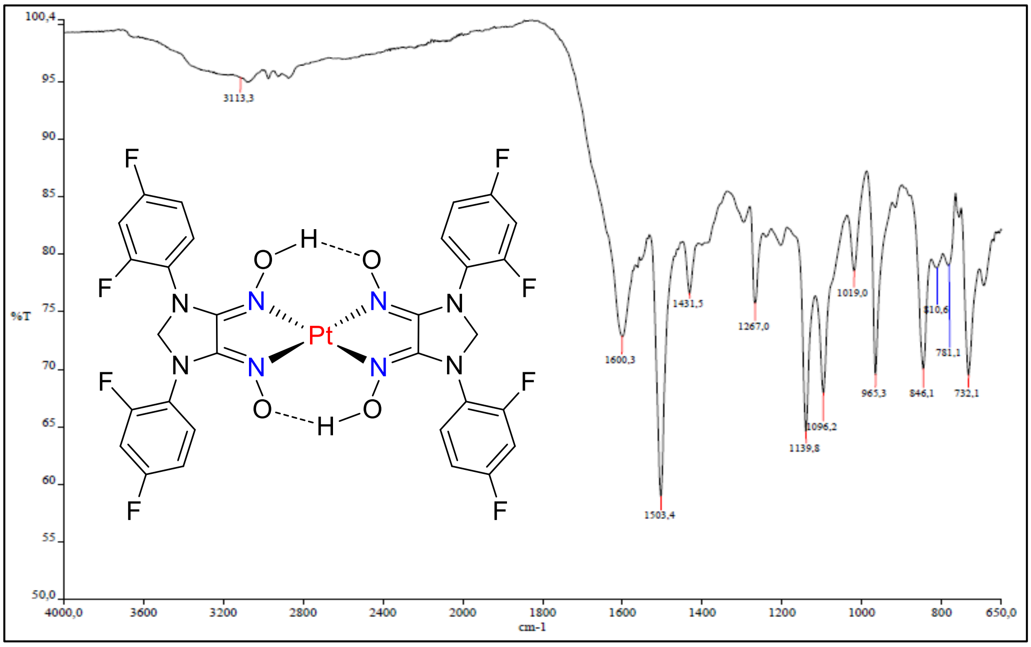

Figure S40. FT-IR spectrum of (4Z,5E)-1,3-bis(2,4-difluorophenyl)imidazolidine-4,5-dionedioxime bis-Pt(II) complex (L_{1c}Pt-b).

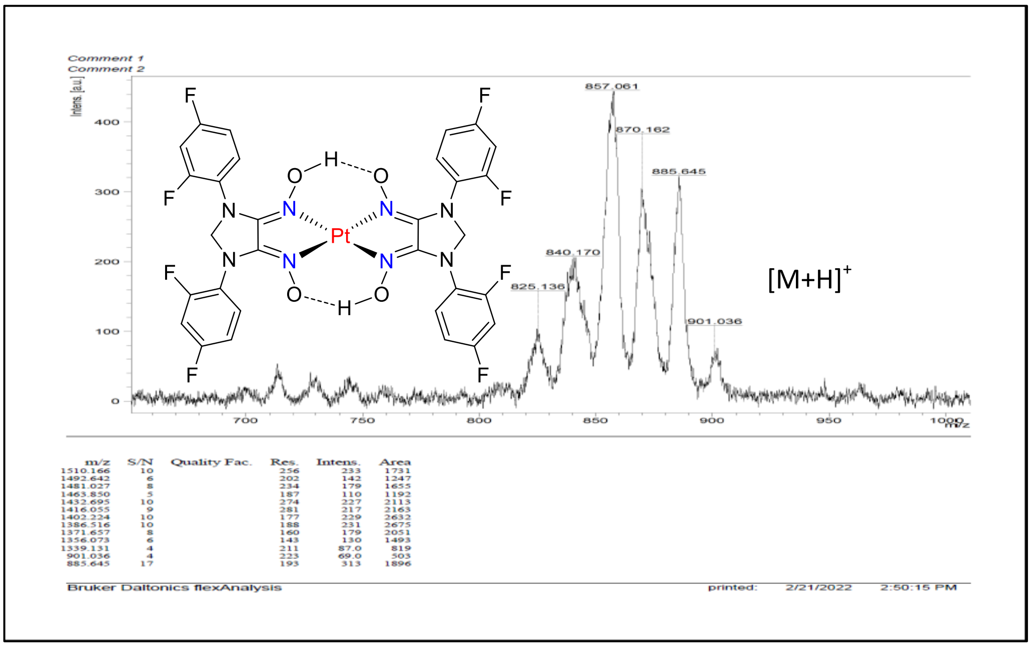

Figure S41. MS spectrum of (4Z,5E)-1,3-bis(2,4-difluorophenyl)imidazolidine-4,5-dionedioxime bis-Pt(II) complex (L_{1c}Pt-b).

3. Materials and methods for biological studies

CCD-1079Sk (CRL-2097), MCF-7 (HTB-22) and MDA-MB-231 (HTB-26) cell lines were used for in vitro studies. MTT (3-(4,5-dimethylthiazol-2-yl)-2,5-diphenyltetrazolium bromide) was purchased from Sigma Aldrich. Dulbecco's modified Eagle's medium/Nutrient Mixture F-12 culture Ham culture media, fetal bovine serum, %0.25 trypsin (1×), Penicillin streptomycin was purchased from Gibco. The apoptosis kit was purchased from BioLegend. The Cell Cycle kit was purchased from Thermo Scientific.

4. Supplementary figures for biological studies

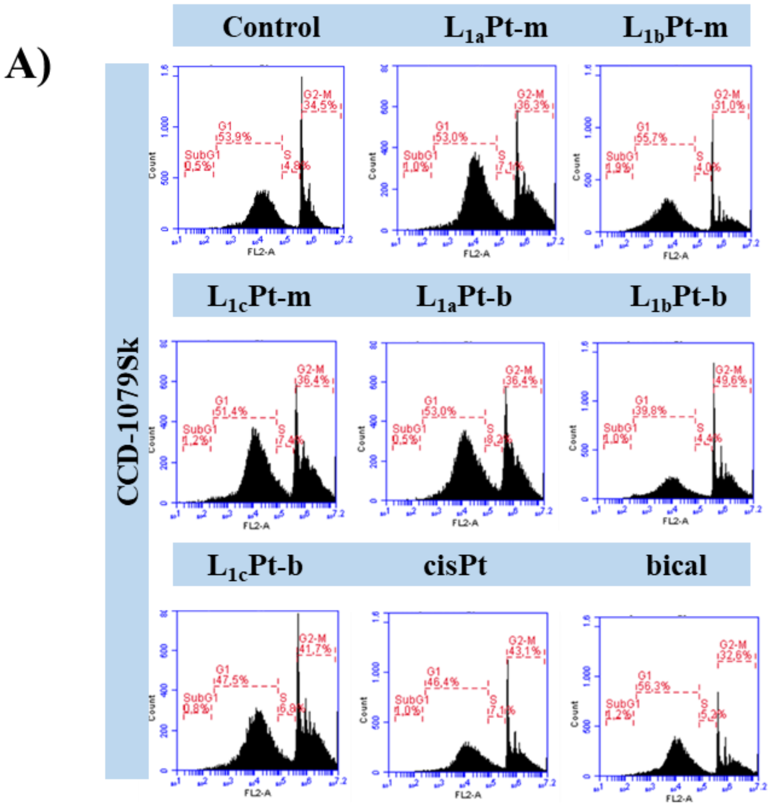

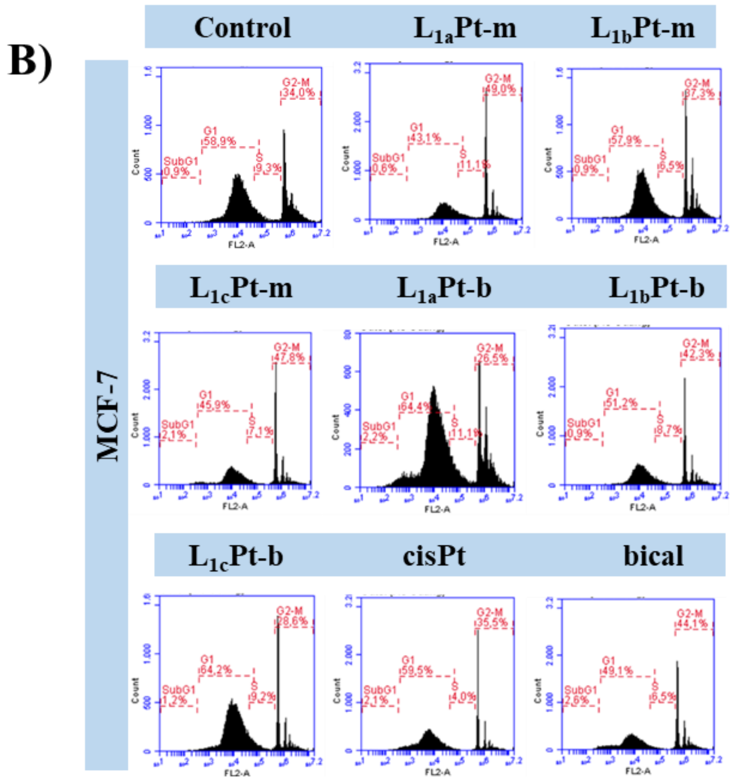

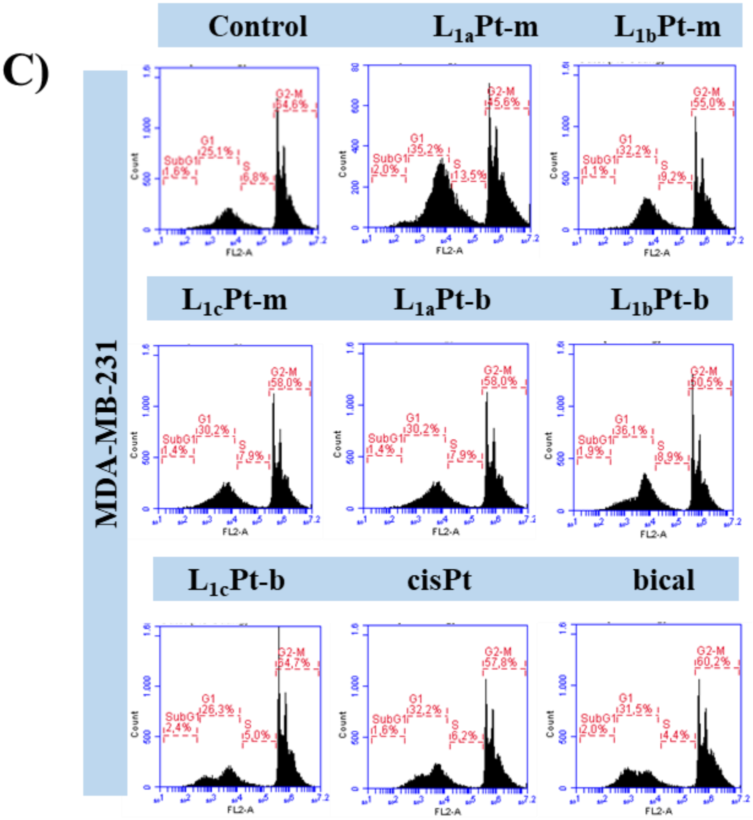

Figure S42. Cell cycle analysis of compounds treated A) CCD-1079Sk, B) MCF-7, and C) MDA-MB-231 cells.

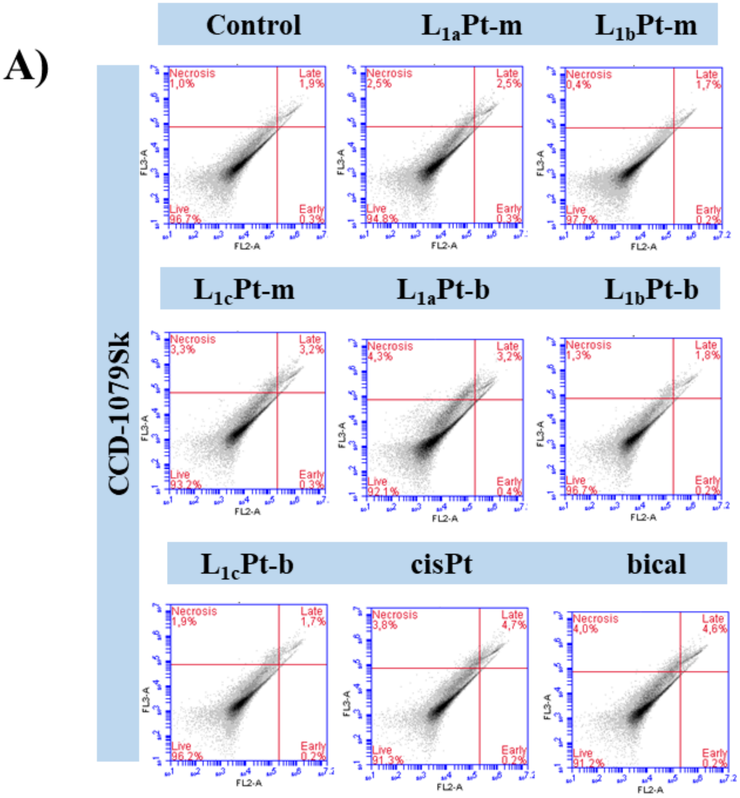
A)
CCD-1079Sk
Control
L1aPt-m
L1bPt-m
L1cPt-m
L1aPt-b
L1bPt-b
L1cPt-b
cisPt
bical
Necrosis 1,0% Late 1,9% Live 96,7% Early 0,3%
Necrosis 2,5% Late 2,5% Live 94,8% Early 0,3%
Necrosis 0,4% Late 1,7% Live 97,7% Early 0,2%
Necrosis 3,3% Late 3,2% Live 93,2% Early 0,3%
Necrosis 4,3% Late 3,2% Live 92,1% Early 0,4%
Necrosis 1,3% Late 1,8% Live 96,7% Early 0,2%
Necrosis 1,9% Late 1,7% Live 96,2% Early 0,2%
Necrosis 3,8% Late 4,7% Live 91,3% Early 0,2%
Necrosis 4,0% Late 4,6% Live 91,2% Early 0,2%
FL3-A
FL2-A

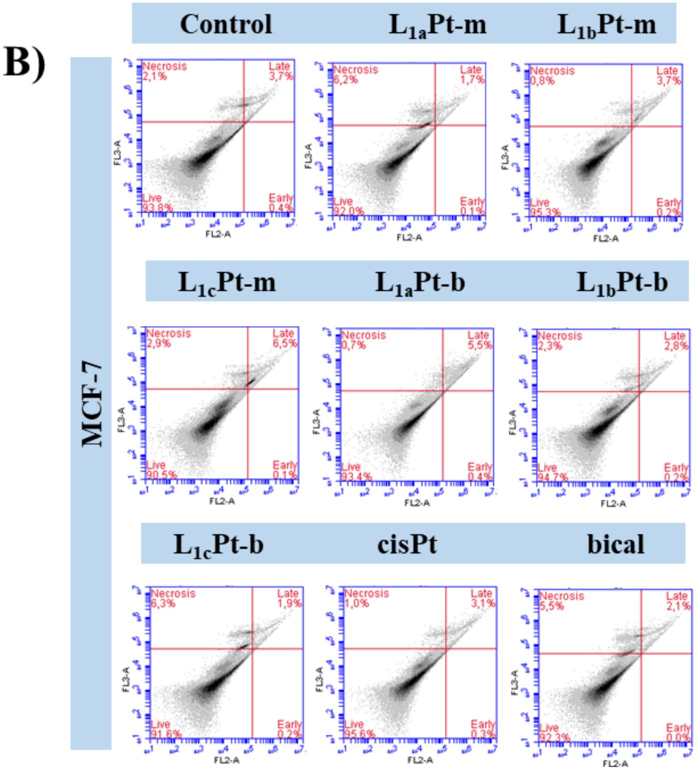
B)
MCF-7
Control
L1aPt-m
L1bPt-m
L1cPt-m
L1aPt-b
L1bPt-b
L1cPt-b
cisPt
bical
Necrosis 2,1% Late 3,7% Live 93,8% Early 0,4%
Necrosis 6,2% Late 1,7% Live 92,0% Early 0,1%
Necrosis 0,8% Late 3,7% Live 95,3% Early 0,2%
Necrosis 2,9% Late 6,5% Live 90,5% Early 0,1%
Necrosis 0,7% Late 5,5% Live 93,4% Early 0,4%
Necrosis 2,3% Late 2,8% Live 94,7% Early 0,2%
Necrosis 6,3% Late 1,9% Live 91,6% Early 0,2%
Necrosis 1,0% Late 3,1% Live 95,6% Early 0,3%
Necrosis 5,5% Late 2,1% Live 92,3% Early 0,0%
FL3-A
FL2-A

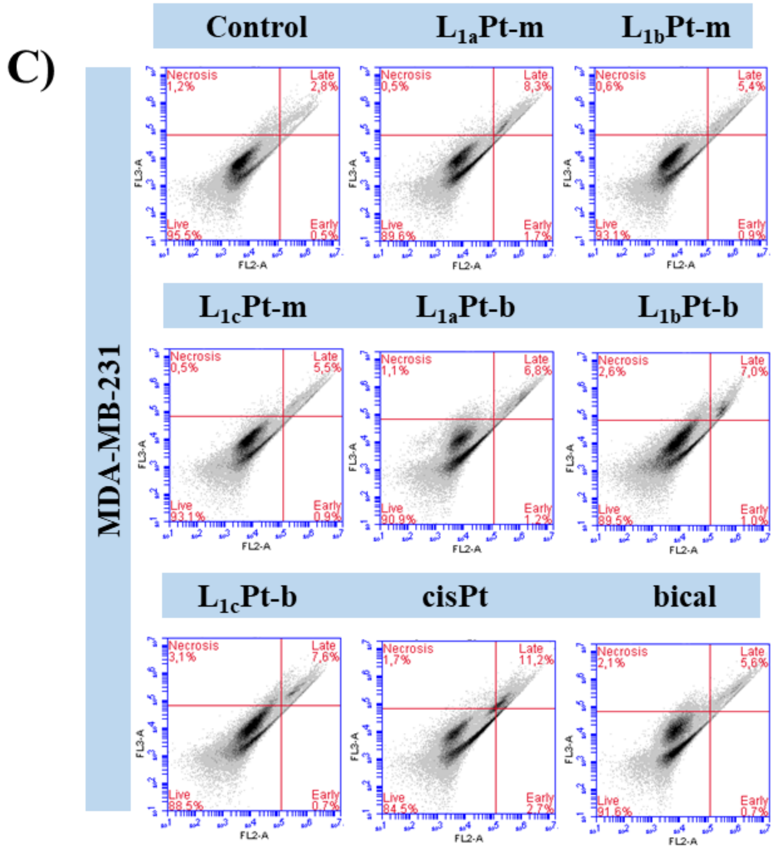

Figure S43. Apoptosis assay results of compounds treated A) CCD-1079Sk, B) MCF-7, and C) MDA-MB-231 cells.

References

[S1] Bruker (2014). APEX2 and SADABS. Bruker AXS Inc., Madison, Wisconsin, USA.

[S2] Bruker (2013). SAINT. Bruker AXS Inc., Madison, Wisconsin, USA.

[S3] Bruker (2014). SADABS, v2014/5, Bruker AXS Inc., Madison, Wisconsin, USA

[S4] Sheldrick GM. SHELXT – Integrated space-group and crystal-structure determination. Acta Crystallographica Section A Foundations and Advances 2015; A71: 3-8. https://doi.org/10.1107/S2053273314026370

[S5] Dolomanov OV, Bourhis LJ, Gildea RJ, Howard JAK, Puschmann H. OLEX2: a complete structure solution, refinement and analysis program. Journal of Applied Crystallography 2009; 42: 339-341. https://doi.org/10.1107/S0021889808042726

[S6] Macrae CF, Edgington PR, McCabe P, Pidcock E, Shields GP et al. Mercury: visualization and analysis of crystal structures. Journal of Applied Crystallography 2006; 39: 453-457. https://doi.org/10.1107/S002188980600731X
